# Air Pollution and Atherosclerosis: A Cross-Sectional Analysis of Four European Cohort Studies in the ESCAPE Study

**DOI:** 10.1289/ehp.1307711

**Published:** 2015-01-27

**Authors:** Laura Perez, Kathrin Wolf, Frauke Hennig, Johanna Penell, Xavier Basagaña, Maria Foraster, Inmaculada Aguilera, David Agis, Rob Beelen, Bert Brunekreef, Josef Cyrys, Kateryna B. Fuks, Martin Adam, Damiano Baldassarre, Marta Cirach, Roberto Elosua, Julia Dratva, Regina Hampel, Wolfgang Koenig, Jaume Marrugat, Ulf de Faire, Göran Pershagen, Nicole M. Probst-Hensch, Audrey de Nazelle, Mark J. Nieuwenhuijsen, Wolfgang Rathmann, Marcela Rivera, Jochen Seissler, Christian Schindler, Joachim Thiery, Barbara Hoffmann, Annette Peters, Nino Künzli

**Affiliations:** 1 Swiss Tropical and Public Health Institute, Basel, Switzerland; 2 University of Basel, Basel, Switzerland; 3 Helmholtz Zentrum München, German Research Center for Environmental Health, Institute of Epidemiology II, Neuherberg, Germany; 4 IUF–Leibniz Research Institute for Environmental Medicine, Düsseldorf, Germany; 5 Unit of Environmental Epidemiology, Institute of Environmental Medicine, Karolinska Institutet, Stockholm, Sweden; 6 Centre for Research in Environmental Epidemiology (CREAL), Barcelona, Spain; 7 CIBER Epidemiologia y Salud Pública (CIBERESP), Spain; 8 Institute for Risk Assessment Sciences, Utrecht University, Utrecht, the Netherlands; 9 Julius Center for Health Sciences and Primary Care, University Medical Center Utrecht, Utrecht, the Netherlands; 10 Environment Science Center, University of Augsburg, Augsburg, Germany; 11 Dipartimento di Scienze Farmacologiche e Biomolecolari, Università di Milano, Milan, Italy; 12 Centro Cardiologico Monzino, IRCCS, Milan, Italy; 13 Cardiovascular Epidemiology and Genetics, IMIM, Barcelona, Spain; 14 Department of Internal Medicine II–Cardiology, University of Ulm, Medical Center, Ulm, Germany; 15 Division of Cardiovascular Epidemiology, Institute of Environmental Medicine, Karolinska Institutet, Stockholm, Sweden; 16 Centre for Environmental Policy, Imperial College London, United Kingdom; 17 German Diabetes Center, Leibniz Center for Diabetes Research at Heinrich Heine University Institute for Biometrics and Epidemiology, Düsseldorf, Germany; 18 University of Montreal Hospital Research Centre (CRCHUM), Montreal, Quebec, Canada; 19 Diabetes Zentrum, Medizinische Klinik und Poliklinik IV–Campus Innenstadt, Klinikum der Ludwig-Maximilians-Universität, München, Germany; 20 Clinical Cooperation Group Diabetes, Ludwig-Maximilians-Universität München and Helmholtz Zentrum München, München, Germany; 21 Institute of Laboratory Medicine, Clinical Chemistry and Molecular Diagnostics, University of Leipzig, Leipzig, Germany; 22 Medical Faculty, Heinrich Heine University of Düsseldorf, Düsseldorf, Germany

## Abstract

**Background:**

In four European cohorts, we investigated the cross-sectional association between long-term exposure to air pollution and intima-media thickness of the common carotid artery (CIMT), a preclinical marker of atherosclerosis.

**Methods:**

Individually assigned levels of nitrogen dioxide, nitrogen oxides, particulate matter ≤ 2.5 μm (PM_2.5_), absorbance of PM_2.5_ (PM_2.5abs_), PM_10_, PM_coarse_, and two indicators of residential proximity to highly trafficked roads were obtained under a standard exposure protocol (European Study of Cohorts for Air Pollution Effects—ESCAPE study) in the Stockholm area (Sweden), the Ausburg and Ruhr area (Germany), and the Girona area (Spain). We used linear regression and meta-analyses to examine the association between long-term exposure to air pollution and CIMT.

**Results:**

The meta-analysis with 9,183 individuals resulted in an estimated increase in CIMT (geometric mean) of 0.72% (95% CI: –0.65%, 2.10%) per 5-μg/m^3^ increase in PM_2.5_ and 0.42% (95% CI: –0.46%, 1.30%) per 10^–5^/m increase in PM_2.5abs_. Living in proximity to high traffic was also positively but not significantly associated with CIMT. Meta-analytic estimates for other pollutants were inconsistent. Results were similar across different adjustment sets and sensitivity analyses. In an extended meta-analysis for PM_2.5_ with three other previously published studies, a 0.78% (95% CI: –0.18%, 1.75%) increase in CIMT was estimated for a 5-μg/m^3^ contrast in PM_2.5_.

**Conclusions:**

Using a standardized exposure and analytical protocol in four European cohorts, we found that cross-sectional associations between CIMT and the eight ESCAPE markers of long-term residential air pollution exposure did not reach statistical significance. The additional meta-analysis of CIMT and PM_2.5_ across all published studies also was positive but not significant.

**Citation:**

Perez L, Wolf K, Hennig F, Penell J, Basagaña X, Foraster M, Aguilera I, Agis D, Beelen R, Brunekreef B, Cyrys J, Fuks KB, Adam M, Baldassarre D, Cirach M, Elosua R, Dratva J, Hampel R, Koenig W, Marrugat J, de Faire U, Pershagen G, Probst-Hensch NM, de Nazelle A, Nieuwenhuijsen MJ, Rathmann W, Rivera M, Seissler J, Schindler C, Thiery J, Hoffmann B, Peters A, Künzli N. 2015. Air pollution and atherosclerosis: a cross-sectional analysis of four European cohort studies in the ESCAPE Study. Environ Health Perspect 123:597–605; http://dx.doi.org/10.1289/ehp.1307711

## Introduction

The cardiovascular effects of air pollution are well recognized (Brook et al. 2010); however, the pathophysiological pathways by which long-term air pollution may affect the cardiovascular system are not completely understood. Experimental and observational studies point to a link between inflammatory processes and the development of atherosclerosis (i.e., atherogenesis) as one of the potential pathways (Libby et al. 2002). The hypothesis that air pollution contributes to atherogenesis through vascular damage due to oxidative stress and systemic inflammation has been supported by animal models (Araujo et al. 2008; Sun et al. 2005; Suwa et al. 2002).

Several epidemiological studies have addressed this hypothesis using measurements of carotid intima-media thickness (CIMT). Cross-sectional measurements of CIMT are an established marker of preclinical stages of atherosclerosis (Lorenz et al. 2012). CIMT is a particularly useful marker to investigate the atherogenic role of ambient air pollution, because it is not sensitive to short-term influences (Künzli et al. 2011). Instead of the binary nature of cardiovascular events, CIMT describes the preclinical and clinical degree of the atherogenic state on a continuous scale. This is of relevance both from a biological perspective to investigate the etiology of the long-term process of atherogenesis and in the context of primary prevention.

So far, only three longitudinal studies (Adar et al. 2013; Künzli et al. 2010; Wilker et al. 2013) in the United States have used CIMT measurements to test the hypothesis of an accelerated progression of CIMT among those with higher cumulative exposure to air pollution and have reported positive associations. These results suggest that chronic exposure to air pollution may accelerate injury to the vasculature. This may lead to a substantial shift in the age of the population at risk of suffering a cardiovascular outcome and may explain stronger associations of mortality based on long-term studies compared with time-series studies (Künzli et al. 2011). A cross-sectional analysis in an adult population should reflect a differential atherogenic progression by an association between measured CIMT and long-term exposure to ambient air pollution.

The first cross-sectional study that tested this hypothesis used data from 798 participants in two clinical trials in Southern California and reported a 4.2% [95% confidence interval (CI): –0.2%, 8.9%] larger CIMT with a 10-μm/m^3^ increase in chronic exposure to PM_2.5_ (Künzli et al. 2005). Several others have also used CIMT data to explore this association (Adar et al. 2013; Bauer et al. 2010; Diez Roux et al. 2008; Erdogmus et al. 2006; Iannuzzi et al. 2010; Lenters et al. 2010; Rivera et al. 2013; Tonne et al. 2012; Wilker et al. 2013). However, the size and direction of associations have varied across studies. In addition to differences in susceptibility or the specific composition or extent of exposures, these inconsistencies might also be a consequence of differences in population measurement of CIMT, statistical models, adjustment sets, or exposure assessment.

The ESCAPE project (European Study of Cohorts for Air Pollution Effects) made unprecedented efforts to standardize the selection, modeling, and assignment of markers of exposure to ambient air pollution, as well as health-related statistical protocols, in a total of 30 European cohorts. Recently published results of prospective analyses of several of these cohorts suggested that particulate matter air pollution contributes to the incidence of coronary events and lung cancer in Europe (Cesaroni et al. 2014; Raaschou-Nielsen et al. 2013). As part of the ESCAPE collaboration, we brought together four established cohorts with available CIMT measurements in adults. The objective of this analysis was to investigate the cross-sectional association between CIMT and a set of markers of long term exposure to ambient air pollution.

## Methods

*Study population and CIMT data collection*. Data from four ongoing European cohort studies were used. IMPROVE-Stockholm (Stockholm, Sweden) is based on 60-year-old adults at recruitment with increased risk for cardiovascular diseases (CVD). KORA (Cooperative Health Research in the Region of Augsburg; Augsburg, Germany), Heinz Nixdorf Recall (HNR; Ruhr Area, Germany), and REGICOR (Registre Gironí del COR; Girona region, Spain) are population-based cohorts (ages 25–75 years at baseline). CIMT was measured at least at one point in time between 1997 and 2009. B-mode ultrasound was used for CIMT measurement in all studies although sonographic protocols differed across studies. Details have been published elsewhere (Baldassarre et al. 2010; Bauer et al. 2009; de Groot et al. 2008; Kowall et al. 2012; Rivera et al. 2013). In brief, in all cohorts, images were obtained by trained sonographers of segments of the left and right common carotid artery at the far artery wall approximately 10 mm proximal to the bulb. In IMPROVE-Stockholm and REGICOR, additional scans were obtained of the carotid bulb, and of the internal carotid 10 mm distal to the flow divider. Although only one image with 45° transducer angle was taken per location for REGICOR and HNR, images at different angles were taken at each location in the other cohorts. CIMT measurement was conducted manually in HNR, in which a maximum of 10 manual CIMT measurements per subject and side were conducted at 0.1-cm intervals over a 1-cm segment. Manual tracing was conducted in REGICOR, but a dedicated scan application protocol was used for CIMT measurements in any given 1 cm of the artery segment. Automatic tracing and measurements were conducted in IMPROVE-Stockholm and KORA. CIMT measurements in HNR were conducted in plaque-free areas only, whereas there was no specific protocol applied regarding plaques in other cohorts (i.e., measurements may include plaques). Only in IMPROVE-Stockholm was the presence of plaques additionally recorded. Cohort population characteristics and CIMT measurements are summarized in Supplemental Material, “Description of cohorts and Carotid-Intima Media Thickness data collection.” For comparability with past studies, and to address differences in CIMT measurement protocols, we used the mean of all IMT measurements of the left and/or right common carotid (CCA) far wall made 10 mm proximal to the bulb as the common outcome for the present analysis.

The four cohorts operate under approval of their respective ethical committees, and all participants gave written informed consent at time of original cohort enrollment.

*Exposure assessment*. We made use of all standard markers of exposure to ambient air pollution developed by the standardized land use regression models (LUR) of ESCAPE (Cyrys et al. 2012; Eeftens et al. 2012). This included different fractions of the particulate matter mass concentrations, PM_2.5_ and PM_10_ (PM with diameter ≤ 2.5 and ≤ 10 μm), the coarse fraction of PM (PM_coarse_), absorbance of PM_2.5_ (PM_2.5abs_), estimates of nitrogen dioxide (NO_2_), and oxides of nitrogen (NO_x_). Two markers of local traffic density were also collected under a standard protocol. Estimates of background levels of NO_x_, and NO_2_ were also available.

Details of standardized ESCAPE protocols and methods used to develop exposure models and traffic markers for each of the four study areas are given elsewhere (Beelen et al. 2013; Eeftens et al. 2012). In brief, particulate matter (PM), NO_x_, and NO_2_ were measured over 2-week periods during three different seasons in 2008–2009 in all four study areas. Measurements were made at about 20 sites for PM and 40 sites for NO_x_ and NO_2_ for the IMPROVE-Stockholm, HNR, and KORA study areas, and at twice as many sites for the REGICOR study area. PM_2.5_ and PM_10_ were collected on preweighed Teflon filters, and PM_coarse_ was obtained as their difference. PM_2.5abs_ was measured on PM_2.5_ filters. Each monitoring site was further characterized by a set of potential geographical predictors. LUR models independently developed at each area were used to explain spatial variation at each measurement site, and the regression models obtained were then used to predict exposure concentrations at each cohort participant’s baseline home address. NO_2_ background LUR models were developed using a similar approach, but the LUR models were based only on regional and urban background sites and background predictors. The performance of the ESCAPE model was routinely tested across all ESCAPE cohorts (Beelen et al. 2013; Eeftens et al. 2012). This was done by first comparing the explained variance between measured and predicted values obtained in the final model at all measured sites (model *R*^2^) and then by comparing measured values and predicted values at all measured sites for a model that was developed by excluding one measurement location at a time [leave-one-out-cross validation (LOOCV) *R*^2^].

The traffic indicators used in ESCAPE are traffic intensity on the nearest road (vehicles × day^–1^) and traffic load on major roads in a 100-m buffer, defined as the sum of traffic intensity multiplied by the length of all major road segments (vehicles × meters × day^–1^). Individual indicators of exposure to traffic were derived from the most recent road networks for Europe and from locally available traffic intensity data (for detailed description, see Supplemental Material, “Exposure assessment methods”).

*Statistical analysis*. We used linear regression to estimate associations between the natural logarithm of CIMT and individually assigned measures of exposure. To independently estimate the effects of living near traffic, we adjusted analyses of traffic indicators for background NO_2_ with associations estimated using exposures modeled as both continuous and categorical variables to facilitate interpretation.

Three predefined adjustment models were used for the main analysis, including a crude model (M1) and a model adjusted by age and sex only (M2). The third model (M3) was adjusted for sex, age and age squared, smoking status (current, former, never/occasional), cigarette pack-years and pack-years squared, education level (low, middle, high), occupational status (employed/self-employed, unemployed, homemaker/housewife, retired), and body mass index (BMI and BMI squared). Covariate definitions were standardized across cohorts to the extent possible. Except for IMPROVE-Stockholm based on two more individuals in M1 and M2 than in M3, for other cohorts, models M1 to M3 were restricted to individuals with complete data for all covariates included in model M3.

For model M3, subgroup analysis was conducted using a set of predetermined variables, namely sex, age (< 60 or ≥ 60 years), BMI (< 30 or ≥ 30 kg/m^2^), education (low, middle, or high), smoking status (current, former, or never/occasional), having either diabetes, impaired fasting glucose (treatment with insulin, oral hypoglycemic drugs or fasting blood glucose > 110 mg/dL) (yes/no), use of antihypertensive medication (yes/no), and use of statins (yes/no). We also hypothesized that clusters of cardiovascular risk factors could interact with exposure to air pollution in complex ways. Therefore, we calculated the Framingham risk score (FRS) for developing a general cardiovascular disease in a 10-year period (Wilson et al. 1998) for each participant and evaluated for effect modification across three predefined levels of risk (low risk, < 10%; moderate risk, 10–20%; and high risk, > 20%). This stratification was also used to facilitate comparison between the older, high-risk IMPROVE-Stockholm cohort and the three younger population-based cohorts, because we assumed that differences among the populations would be less pronounced within strata defined by FRS categories. We further evaluated differences in effects between long-term residents and short-term residents. Long-term residents were defined as subjects living at the same address ≥ 10 years. For the HNR study, residential history was not available for all participants, and 5 years was the longest available cut-off. Thus HNR was excluded from this sub-analysis.

Three additional stepwise adjustment models were developed for sensitivity analyses. First, we additionally adjusted model M3 by physical activity (categorized as low, middle, or high, or according to metabolic equivalents, depending on availability), alcohol intake (categories of drinks per week), and wine consumption (model M4a). Model M4a was further adjusted for continuous levels of systolic blood pressure and high- and low-density lipoprotein (HDL and LDL) (model M4b). Model 5 was adjusted for covariates in model M4b plus antihypertensive and statin medication use (M5). All covariates were defined *a priori*.

We additionally assessed the sensitivity of results by using estimates of air pollution back-extrapolated to the year of the CIMT measurements; adjusting for long-term noise exposure in 5-dB categories of day-evening-night noise (*L*_den_) or night noise (*L*_night_); and by accounting for potential clustering by area because individuals living in the same areas may share similar characteristics (e.g., socioeconomic and environmental). ESCAPE exposure concentrations were developed with data collected between 2008 and 2009 that do not correspond to the year of CIMT measurement at each cohort. To adjust for possible differences in air pollution levels between time points and given the lack of historic LUR models to reconstruct historic spatial trends, individual exposures were back-extrapolated as follows: In each study region, available historic annual means (NO_2_, NO_x_, and PM_10_ only) from fixed-site monitoring stations were used to calculate the ratio between the average annual concentrations for the period of interest in the past and the period of the ESCAPE measurement. Individual ESCAPE exposure for each study participant was then multiplied by this ratio. Detail of the back-extrapolated approach followed in ESCAPE has been described elsewhere (Cesaroni et al. 2012). Although this approach was meant to capture the long-term general changes in urban background pollution, it did not account for potential spatial within-city individual exposure changes. Exposure to ambient noise was obtained from the first round of noise mapping developed in the European Union (EU) in 2007 following the 2002 EU directive that required that all member states produce every fifth year a noise map for major roads, major railways, and major airports and for larger agglomeration (European Commission 2002). To control clustering by area, a maximum-likelihood random-effects model was used. Area level was represented by an indicator of the neighborhood for IMPROVE-Stockholm and HNR, an indicator of municipality for REGICOR, and by a 5 × 5 km grid indicator for KORA.

Cohort-specific results were meta-analyzed for both fixed and random-effects and reported in forest plots. The heterogeneity of effect estimates among studies was evaluated with the *I*^2^ statistic (Higgins and Thompson 2002). In the absence of heterogeneity, results from fixed-effects models are reported when describing the results. In the case of significant heterogeneity (*p* < 0.1 or *I*^2^ > 50%), random effects are reported instead (DerSimonian and Laird 1986). Because the meta-analyses were based on only four individual studies, we did not attempt to evaluate the influence of specific study characteristics on the summary estimates. Subgroup-specific estimates were also meta-analyzed. Differences in stratum-specific effect estimates were qualitatively evaluated, without any formal test of the interactions.

In an expanded meta-analysis, ESCAPE estimates for PM_2.5_ were combined with estimates from other published cross-sectional studies that also used CIMT as outcome. We used a previous review to identify relevant studies (Rivera et al. 2013) and also searched PubMed (http://www.ncbi.nlm.nih.gov/pubmed) to identify any additional studies published online before 2 September 2013. Different combinations of the key words “intima media thickness,” “air pollution,” “fine particulate air pollution,” “progression,” and ”atherosclerosis” were used in the search strategy.

All statistical analyses were conducted using Stata (version 12.1; StataCorp, College Station, TX, USA). Results are presented for a preselected set of exposure contrasts that cover the variability of exposures observed across the ESCAPE project. The exposure contrasts for descriptive and categorical association analyses of traffic indicators were chosen to facilitate the interpretation of results throughout the ESCAPE project. For example, for traffic intensity at the nearest road, we used a 5,000 vehicle per day contrast, which is approximately equal to the traffic density of many urban roads in Europe, and thus represents the effect of a doubling of the traffic intensity on a typical major road. The default alpha level for statistical significance was assumed as 0.05.

## Results

A total of 9,183 individuals were included in our study (based on a complete case analysis for model M3). Depending on the cohort, this represented 78–87% of the total cohort participants with both valid CIMT and air pollution measurements. A summary of common individual characteristics is provided in Table 1. Mean CIMT ranged from 0.68 mm (in HNR) to 0.85 mm (in IMPROVE-Stockholm and KORA). Because of selection for higher cardiovascular risk, IMPROVE-Stockholm participants were older and more likely to be diabetic, and had lower levels of HDL and higher blood pressure on average than participants in the other cohorts. In addition, although participants from IMPROVE-Stockholm were less likely to be current smokers, they were more likely to be former smokers. Reported use of lipid-lowering medication was considerably more prevalent in REGICOR than in any other cohort. Educational levels differed considerably across cohorts. For example, 8% of participants were classified as having low education in KORA compared with 51.4% in REGICOR.

**Table 1:** Distribution of CIMT and selected baseline individual characteristics in the four cohort studies contributing to this ESCAPE analysis.

Characteristic	IMPROVE-Stockholm	HNR	KORA	REGICOR
*n*^*a*^	487	3,759	2,646	2,291
Geographic location	Stockholm area (Sweden)	Ruhr area (Germany)	Augsburg (Germany)	Girona area (Spain)
Year of CIMT measurements	1997–1999	2001–2003	2006–2008	2007–2009
CIMT (mm)	0.85 ± 0.16	0.68 ± 0.13	0.85 ± 0.14	0.70 ± 0.15
Women (%)	50.0	51.0	52.0	55.0
Age (mean ± SD)	66.8 ± 0.38	59.7 ± 7.8	55.8 ± 13.0	58.5 ± 12.2
Body mass index (mean ± SD)	26.8 ± 4.1	27.9 ± 4.6	27.7 ± 4.8	26.8 ± 4.3
Educational level (%)
Low	24.1	10.9	8.1	51.4
Middle	49.1	55.3	76.2	28.6
High	26.1	33.9	15.8	20.0
Occupational status (%)
Employed/self-employed	55.0	40.3	51.9	52.9
Unemployed	10.1	13.7	2.0	2.6
Homemaker/housewife	7.4	39.7	10.3	13.0
Retired	27.5	6.3	35.9	31.5
Smoking status (%)
Current	12.3	23.2	18.6	16.4
Former	41.3	35.3	38.7	27.0
Never or occasional	46.4	41.5	42.6	56.6
Total pack-years in current/former smokers (mean ± SD)	11.2 ± 15.5	15.63 ± 24.8	11.6 ± 19.2	23.93 ± 11.9
Wine drinks per week (mean ± SD)	5.08 ± 7.8	5.42 ± 10.5	4.04 ± 7.8	4.23 ± 7.7
Physical activity in metabolic equivalents (mean ± SD)	NA	1,131 ± 2,110	NA	2,009 ± 1,926
Physical activity (%)
Low	10.5	NA	31.8	NA
Medium	54.4	NA	44.0	NA
High	35.1	NA	24.2	NA
LDL (mg/dL)	139.1 ± 37.1	146.5 ± 36.2	136.3 ± 34.8	137.7 ± 31.8
HDL (mg/dL)	49.7 ± 14.7	57.9 ± 17.2	56.1 ± 14.5	54.7 ± 12.4
Diastolic blood pressure (mmHg)	84.8 ± 9.3	81.1 ± 10.7	75.1 ± 9.9	77.4 ± 10.1
Systolic blood pressure (mmHg)	149.8 ± 19.1	132.6 ± 20.6	122.2 ± 18.1	126.4 ± 18.7
Lipid-lowering medication (yes) (%)	27.5	10.3	11.4	39.3
Diabetes^*b*^ (yes) (%)	16.0	13.4	7.4	12.6
Hypertensive medication (yes) (%)	47.8	35.6	29.9	24.0
NA, not available for the cohort. ^***a***^Based on complete case analysis for model M3. ^***b***^Defined as impaired fasting glucose (blood glucose level > 110 mg/dL) or treatment with insulin or oral hypoglycemic drugs.				

The distribution of air pollution exposures by cohort is presented in Table 2. Mean levels of PM_2.5_ varied between 7.2 and 18.4 μg/m^3^, between 0.6 and 2.1 10^–5^/m for PM_2.5abs_, between 14.7 and 30.8 μg/m^3^ for PM_10_, between 6.2 and 15.6 μg/m^3^ for PM_coarse_, between 10.4 and 32.5 μg/m^3^ for NO_2_, and between 18.1 and 56.1 μg/m^3^ for NO_x._ The lowest mean levels of pollutant exposures, except for PM_coarse_, were estimated for participants in IMPROVE-Stockholm. Apart from PM_2.5_, mean exposures, including the traffic indicators, were highest in REGICOR (Table 2). For REGICOR, < 57% of individuals lived in the lowest categories of traffic intensity and traffic load, whereas this percentage was > 65% for the other cohorts (see Supplemental Material, Table S1). With a few exceptions, exposure contrasts, indicated by the interquartile ranges (IQRs), were very small for PM in all cohorts (e.g., for PM_2.5_ the IQR ranges between 1.1 and 1.7 μg/m^3^) but rather large for NO_2_ or NO_x_ (e.g., for NO_2_ the IQR ranges between 3.7 and 17.8 μg/m^3^) (Table 2).

**Table 2:** Summary of cohort-specific individually assigned air pollutant and traffic exposure indicators

Cohort/pollutant indicator	Mean ± SD	Minimum	Median	Maximum	IQR
IMPROVE-Stockholm (*n* = 487)
PM_2.5_ (μg/m^3^)	7.2 ± 1.3	4.2	7.3	10.8	1.7
PM_2.5abs_ (10^–5^/m)	0.6 ± 0.2	0.4	0.6	1.3	0.1
PM_coarse_ (μg/m^3^)	7.1 ± 3.0	0.7	7.4	20.3	3.0
PM_10_ (μg/m^3^)	14.7 ± 4.0	6.0	15.1	31.1	4.1
NO_2_ (μg/m^3^)	10.4 ± 4.1	6.0	9.1	31.1	3.7
NO_x_ (μg/m^3^)	18.1 ± 8.9	11.4	14.6	73.3	6.0
Traffic intensity at the nearest road (vehicles × day^–1^ × 10^–4^)	0.15 ± 0.33	0.02	0.05	2.9	0.05
Traffic load within 100 m on major roads (vehicles × day^–1^ × m^–1^ × 10^–4^)	54.2 ± 180.5	0.0	0.0	2620.0	0.0
HNR (*n* = 3,759)
PM_2.5_ (μg/m^3^)	18.4 ± 1.1	16.0	18.3	21.4	1.5
PM_2.5abs_(10^–5^/m)	1.6 ± 0.3	1.0	1.5	3.4	0.4
PM_coarse_ (μg/m^3^)	10.0 ± 1.8	0.8	10.1	15.0	1.9
PM_10_ (μg/m^3^)	27.8 ± 1.8	23.9	27.5	34.5	2.1
NO_2_ (μg/m^3^)	30.3 ± 4.9	19.8	29.6	62.4	6.3
NO_x_ (μg/m^3^)	50.9 ± 11.9	24.3	49.7	120.0	16.3
Traffic intensity at the nearest road (vehicles × day^–1^ × 10^–4^)	NA	NA	NA	NA	NA
Traffic load within 100 m on major roads (vehicles × day^–1^ × m^–1^ × 10^–4^)	109.6 ± 221.0	0.0	0.0	2682	145.5
KORA (*n* = 2,646)
PM_2.5_ (μg/m^3^)	13.6 ± 0.9	11.8	13.5	17.8	1.1
PM_2.5abs_ (10^–5^/m)	1.7 ± 0.2	1.3	1.7	2.6	0.2
PM_coarse_ (μg/m^3^)	6.2 ± 1.1	4.1	6.1	12.6	1.2
PM_10_ (μg/m^3^)	20.4 ± 2.4	14.8	20.5	30.7	3.2
NO_2_ (μg/m^3^)	18.8 ± 3.8	11.5	18.4	39.1	5.0
NO_x_ (μg/m^3^)	32.8 ± 7.3	19.7	31.4	75.2	8.8
Traffic intensity at the nearest road (vehicles × day^–1^ × 10^–4^)	0.16 ± 0.32	0.0	0.05	3.3	0.0
Traffic load within 100 m on major roads (vehicles × day^–1^ × m^–1^ × 10^–4^)	41.5 ± 103.7	0.0	0.0	1177.0	0.0
REGICOR (*n* = 2,291)
PM_2.5_ (μg/m^3^)	14.9 ± 1.6	9.0	14.9	21.3	1.3
PM_2.5abs_ (10^–5^/m)	2.1 ± 0.7	1.1	2.0	4.5	0.8
PM_coarse_ (μg/m^3^)	15.6 ± 2.7	9.9	14.9	26.4	3.7
PM_10_ (μg/m^3^)	30.8 ± 4.9	20.8	30.1	47.2	5.8
NO_2_ (μg/m^3^)	32.5 ± 12.0	10.1	33.0	78.7	17.8
NO_x_ (μg/m^3^)	56.1 ± 24.2	15.3	55.4	175.0	31.4
Traffic intensity at the nearest road (vehicles × day^–1^ × 10^–4^)	0.34 ± 0.57	0.0	0.11	3.4	0.30
Traffic load within 100 m on major roads (vehicles × day^–1^ × m^–1^ × 10^–4^)	127.0 ± 199.5	0.0	0.0	1013.0	207.1
NA, not available for the cohort.					

Patterns of correlations between pollutants varied considerably across cohorts (see Supplemental Material, Table S2). For example the Spearman correlation coefficient (*r*) between PM_2.5_ and NO_2_ was around 0.6 in IMPROVE-Stockholm, HNR, and REGICOR, but only 0.38 in KORA. Similarly, a low *r* was observed between PM_2.5_ and PM_2.5abs_ in this cohort (0.44), although it was > 0.8 in others. Correlation coefficients between pollutants and traffic indicators were low to moderate (0.08–0.62). Previously published *R*^2^ for model validation ranged across pollutants from 62% to 90% and from 51% to 87% for LOOCV *R*^2^ (see Supplemental Material, Table S2). The difference between model *R*^2^ and LOOCV *R*^2^ never exceeded 19% (percent point), below the 20% threshold usually interpreted as indication of potential model bias (Eeftens et al. 2012).

In cohort-specific analyses of long-term air pollution exposures and CIMT, there were no statistically significant positive associations based on adjusted models (models M2–M5) except for positive associations with PM_2.5_ in KORA and PM_2.5abs_ in REGICOR (both for model M2 only) (see Supplemental Material, Figure S1). In IMPROVE-Stockholm, a pattern of inverse associations was seen across all exposures, reaching statistical significance for PM_10_, PM_coarse_, NO_2_, and NO_x_ (see Supplemental Material, Figure S1A). Associations between traffic load and/or intensity were inconsistent between HNR and KORA and IMPROVE-Stockholm and REGICOR (see Supplemental Material, Figure S1E,F). Only for the latter cohorts did estimates with traffic load reach statistical significance in model M3. For all pollutants, in general, results were robust to the different adjustment sets, although models M4a, M4b, and M5 were based on fewer participants because of missing covariate data.

**Figure 1:**
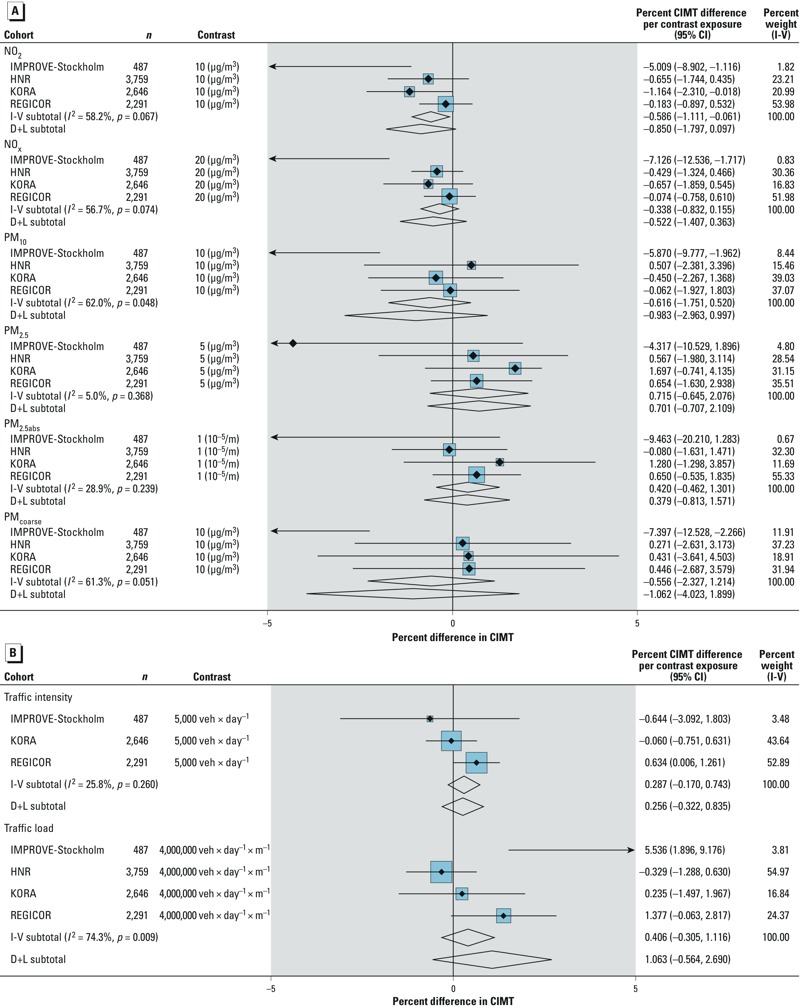
Forest plot of the percent difference in CIMT (geometric mean with 95% CIs) for model M3 for (*A*) ESCAPE air pollutants per standard contrast of exposure as indicated in the figure, and (*B*) ESCAPE continuous traffic indicators. Traffic intensity: at the nearest road per contrast of exposure of 5,000 vehicles (veh) × day^–1^. Traffic load: within 100 m on major roads per contrast of exposure of 4,000,000 vehicles (veh) × day^–1^ × m^–1^. Fixed (I-V subtotal) and random effects [D+L (DerSimonian and Laird method)] are shown. *I*^2^: variation in estimated effects attributable to heterogeneity with percent weight I-V (inverse variance) as relative percent weight of each cohort (blue boxes). For IMPROVE-Stockholm, the arrow indicates direction of the effect estimate. Model M3 was adjusted for sex, age (centered on the sample mean), age^2^, smoking status (3 categories), smoking pack-years (centered), smoking pack-years^2^, education level (3 categories), occupation status (4 categories), BMI (centered), BMI^2^, indicator of city residence when applies.

Meta-analytic model M3 estimates of the association between CIMT and air pollution levels using ESCAPE cohort-specific estimates are presented in Figure 1A. Summary estimates across the four cohorts (*n* = 9,183) were positive but not statistically significant for PM_2.5_ and PM_2.5abs_. The combined fixed-effects estimates indicated a 0.72% (95% CI: –0.65%, 2.1%) increase in CIMT (geometric mean) per 5-μg/m^3^ increase in PM_2.5_ and a 0.42% (95% CI: –0.46%, 1.30%) increase per 10^–5^/m increase in PM_2.5abs_. Summary estimates for the other pollutants (PM_coarse_, PM_10_, NO_2_, and NO_x_) were inverse but not statistically significant, though there was significant heterogeneity across the studies (*I*^2^ > 50% or *p* < 0.1) in associations with all four pollutants. Estimates from combined analyses without IMPROVE-Stockholm, that showed a pattern of inverse significant results for these pollutants, did not change (result not shown) except for PM_coarse_, for which direction of effects changed although remained non-statistically significant (0.37%; 95% CI: –1.49%, 2.26%).

We found positive but not statistically significant associations for traffic indicators (Figure 1B). For example, when considered on a continuous scale, we found a fixed-effects estimate of 0.29% (95% CI: –0.17%, 0.74%) higher CIMT (geometric mean) per 5,000 vehicles × day^–1^ in traffic intensity (over three cohorts only) and a 1.1% (95% CI: –0.56%, 2.7%) increase per 4,000,000 vehicles × day^–1^ × m^–1^ of traffic load (reported as random-effects because of significant heterogeneity). Estimates by categories of traffic markers were similarly positive but with some inconsistency across categories given the inhomogeneous distribution of traffic counts between cohorts. For example, for traffic load estimates were only generated for the third and fourth categories, and categorical associations for traffic intensity were positive for the second and fourth categories but null for the third (see Supplemental Material, Table S3).

Meta-analytic estimates did not differ materially when adjusted for a random effect for neighborhood or when adjusted for noise (*L*_den_ or *L*_night_; results not shown for the latter) (see Supplemental Material, Table S4). Results remained similar when correcting exposures for historical trends (available only for NO_2_, NO_x_, and PM_10_).

Subgroup-specific meta-analytic results are illustrated in Supplemental Material, Figure S2, for three selected pollutants. Some differences in magnitude of stratum-specific associations are worth mentioning: Associations appeared to be stronger in current smokers than in former smokers or nonsmokers (all pollutants); for NO_x_, all subgroup meta-analysis remained inversely non-statistically significant; for PM_2.5_, effects remained positive only for younger people, non-obese, non-diabetics, those with intermediate/higher education level, those using statin medication, and those with an intermediate FRS. Sex and use of hypertensive medication did not materially modify the direction of the main effects, and inverse association was observed for both long-term and short-term residents. For PM_2.5abs_ inverse associations for males and those with low FRS were observed. Effects remained positive for both long-term and short-term residents.

**Figure 2:**
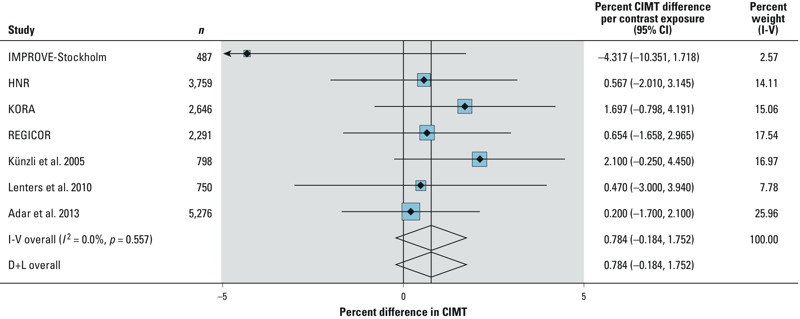
Forest plot of the percent difference in CIMT (geometric mean with 95% CIs) per 5 μg/m^3^ PM_2.5_ using the four ESCAPE cohort and previously published results. Fixed (I-V subtotal) and random effects [D+L (DerSimonian and Laird method)] are shown. *I*­^2^: variation in estimated effects attributable to heterogeneity with percent weight I-V (inverse variance) as relative percent weight of each cohort (blue boxes). For IMPROVE-Stockholm arrow indicates direction of the effect estimate. Estimates of ESCAPE cohorts based on model M3 adjusted for: sex, age (centered on the sample mean), age^2^, smoking status (3 categories), smoking pack-years (centered), smoking pack-years^2^, education level (3 categories), occupation status (4 categories), BMI (centered), BMI^2^, indicator of city residence when applies. Other adjustment sets: for Künzli et al. (1995): sex, education, income, active and passive smoking, multivitamins, alcohol intake (Table 2); for Lenters et al. (2010): age, sex, pulse pressure, BMI, pack-years of smoking, parental smoking at home during childhood, alcohol intake, education, highest profession, diabetes, and percent of low and high income households in neighborhood (Table 2); for Adar et al. (2013): sex, age ethnicity, education, neighborhood socioeconomic score, adiposity, pack-years at baseline, and time-varying smoking status (Table 2).

We identified three studies reporting on a cross-sectional association between CIMT and PM_2.5_ suitable to be included in an extended meta-analysis. Two studies were conducted on populations > 40 years of age (Adar et al. 2013; Künzli et al. 2010), whereas the other study population was approximately 25 years of age on average (Lenters et al. 2010). Previously published results of HNR (Bauer et al. 2010) were not retained, because this cohort was included in the primary ESCAPE analysis. We used the most recent cross-sectional results reported for the Multi-Ethnic Study of Atherosclerosis and Air Pollution (MESA) population (Adar et al. 2013). Given the very young age, we discarded one cross-sectional study of nonsmoking high-school students in the United States (Breton et al. 2012). Exposure assessment in a study by Lenters et al. (2010) was based on a similar LUR approach used in ESCAPE; Künzli et al. (2010) used a geostatistical model to derive exposure assessment. MESA was based on a spatiotemporal model that also incorporated a component of LUR to predict concentrations at locations and times where measurements were not available (Cohen et al. 2009). In the study by Künzli et al. (2010) and the MESA study, only measurements from the right common carotid were examined. Using results from models similar to our model M3, the extended meta-analytic estimate indicated a 0.78% (95% CI: –0.18%, 1.75%, *p* = 0.11) difference in CIMT per 5-μg/m^3^ contrast in PM_2.5_ (Figure 2). For the population-weighted mean CIMT of 0.743 mm across the four ESCAPE cohorts, this result would correspond to a mean difference in CIMT of 5.8 μm with a 5-μg/m^3^ increase in PM_2.5_. No evidence for heterogeneity was observed (*I*^2^ = 0% or *p* = 0.557).

## Discussion

In a meta-analyses of four cross-sectional European studies, we found positive but not statistically significant associations between CIMT and long-term estimates of residential exposure to several markers of air pollution, namely PM_2.5_, PM_2.5abs,_ traffic load within 100 m of home, and traffic intensity at the nearest road. In contrast, inverse non-statistically significant associations were estimated for NO_2_, NO_x_, PM_10_, and PM_coarse_. It is a major strength of ESCAPE that fully standardized sets of exposure metrics were derived to allow comparability across cohorts that otherwise present substantial population heterogeneity. Other strengths of this study include assessment of a comprehensive set of pollutants, cohorts covering a wide range of exposures, large numbers of participants, common information about potential confounders, and comparability of health analysis methods.

Except for IMPROVE-Stockholm, our cohort-specific and combined ESCAPE estimates for PM_2.5_ were within the range of other cross-sectional studies. A 5-μg/m^3^ increase in PM_2.5_ was associated with a 2.1% (95% CI: –0.1%, 4.4%) higher CIMT among older adults in Los Angeles, California (Künzli et al. 2005). A 0.47% (95% CI: –3.0%, 3.94%) increase of CIMT per 5-μg/m^3^ PM_2.5_ contrast was reported in the population-based study Atherosclerosis Risk in Young Adults, conducted in the Netherlands (Lenters et al. 2010). In Germany, associations between PM_2.5_ and CIMT were slightly larger (4.1% increase; 95% CI: 1.7%, 6.5%, per 4.2 μg/m^3^ PM_2.5_), based on an earlier analysis of the HNR study using a different exposure model (Bauer et al. 2010). In MESA, a 5-μg/m^3^ increase in PM_2.5_ within cities was associated with a 0.2% (95% CI: –1.7%, 2.1%) increase in CIMT based on a model similar to our model M3. When these existing cross-sectional studies—except HNR, to avoid including the same study population twice—and our ESCAPE estimates were combined, the estimated difference in CIMT with a 5-μg/m^3^ increase in PM_2.5_ was < 1%.

In addition to PM_2.5_, our ESCAPE summary estimates were positive only for the set of standardized traffic indicators and PM_2.5abs_. The literature does not provide comparable estimates to expand the meta-analysis to these markers. PM_2.5abs_ is considered a better marker of traffic-related particles than PM_2.5_, partly because of its larger spatial heterogeneity. Only one other study has used this indicator to evaluate the association between CIMT and long-term exposure to air pollution (Wilker et al. 2013). Despite a very different population (elderly men only), this study conducted in the greater Boston, Massachusetts, area reported that a spatially resolved estimate of the home outdoor 1-year average black carbon concentration was associated with a 1.1% higher CIMT (95% CI: 0.4%, 1.4%) per 0.26-μg/m^3^ increase of this pollutant. Our results for PM_10_ were fairly inconsistent with those from a study based on 2,348 participants of the Whitehall II cohort of British civil servants and from a past HNR study (Bauer et al. 2010; Tonne et al. 2012). Whitehall II reported a 5% difference (95% CI: 1.9%, 8.3%) for an IQR increase of 5.2 μg/m^3^ PM_10_. HNR reported a positive though not statistically significant association with PM_10_ (1.8% change; 95% CI: 0.6%, 4.3%, per 6.7 μg/m^3^ PM_10_).

Our effect estimates were robust to several tests. The internal validation was good for the exposure models developed for our four cohorts. Adding covariates that may be on the causal pathway linking air pollution with atherosclerosis, such as blood pressure or medication to control blood pressure, did not substantially attenuate the coefficients. Associations also were not confounded by noise. Estimates were robust to adjustment for potential clustering by area, although the indicators used in the different cohorts represented different spatial dimensions, and residual confounding by area cannot be ruled out. We had no true long-term estimates of exposure, so the analyses rely on the assumption that current levels, as estimated in ESCAPE during 2008–2009, reflect long-term exposures before the CIMT measurement. However, the similarity of associations among long-term residents compared with those who moved residence (see Supplemental Material, Figure S2) suggests limited sensitivity. Studies investigating the validity of LUR-modeled exposures also suggest that the ESCAPE-modeled exposure reflects the spatial contrasts reasonably well over years (Cesaroni et al. 2012).

It has been hypothesized that long-term air pollution exposure could act through a pathophysiological pathway that leads to endothelial dysfunction and subclinical atherosclerosis (Brook and Rajagopalan 2010). In a study in Los Angeles, CIMT progression was estimated to be accelerated by 0.6 μm/year (95% CI: –0.1, 1.4 μm/year) per 2.5 μg/m^3^ PM_2.5_ (Künzli et al. 2010). For the participants of the MESA population conducted in six cities across the United States, a 5.0-μm/year (95% CI: 2.6, 7.4 μm/year) faster progression of CIMT per 2.5 μg/m^3^ PM_2.5_ was estimated (Adar et al. 2013). Atherosclerosis is the underlying cause for many cardiovascular outcomes. If air pollution accelerates atherosclerosis, the burden of air pollution may be larger than anticipated. By extension, a reduction of long-term exposure to air pollution may result in delays or reduction of this burden (Künzli et al. 2011). It is possible that CIMT does not reflect the differential lifelong processes of atherosclerosis in different vascular beds and especially plaque formation in the carotid artery, which is more strongly related with clinical endpoints (Lorenz et al. 2012). Development of atherosclerosis, together with interactions with other biological pathways or added susceptibility to acute air pollution triggers, could help explain such large risk as well (Brook and Rajagopalan 2010).

Our study presents weaknesses which may in part explain the null findings. The cross-sectionally assessed CIMT may be the result of all cumulative past atherogenic and atheroprotective exposures, including but not limited to air pollution (Künzli et al. 2011). In addition, because exposure contrasts were rather limited within studies for most markers of exposure, statistical power to detect significant effects in such settings may be limited. The protocols and methods to measure CIMT differed across cohorts, though all studies tested the internal validity of their CIMT measurement methods and protocols. For example, high intra- and interobserved repeatability measures have been reported (Baldassarre et al. 2007; Bauer et al. 2009; Kowall et al. 2012; Rivera et al. 2013). Our study design did not permit comparisons of validity across studies. However, the standardized analytical approach followed in ESCAPE aimed to minimize the possibility that large systematic bias has occurred.

The four studies had different designs and protocols for covariate assessments, thus, there were only limited options to more precisely operationalize some of these covariates in ways that would still be consistent across the studies. For example, socioeconomic status could only be represented by three levels of education and unspecific occupational status in the minimum adjusted model (model M3).

Current smokers had stronger risk estimates, especially with PM_2.5_ exposure. Others have hypothesized that the difference in the precision of CIMTs measurements or competing risks for CIMT progression in some susceptible populations can bias results (Adar et al. 2013; Rivera et al. 2013). The stratification by the FRS showed that when populations were made similar across cohorts, no modification existed. Thus modification by susceptibility factors such as smoking status could be interpreted here as an indication of some difference by location and may in part relate to the exposure modeling approach.

Finally, nonsystematic exposure misclassification is a potential cause of bias toward null findings. Two of our cohorts previously published estimates of cross-sectional associations between CIMT and pollution based on other exposure models, but using data from most of the same subjects (Bauer et al. 2010; Rivera et al. 2013). In REGICOR, individual exposure to NO_2_ was estimated as the 10-year time-weighted average of assigned home outdoor concentrations. The local REGICOR LUR model was based on 562 NO_2_ measurements in Girona and the 10 surrounding communities where participants lived (Rivera et al. 2013). The difference in number of sampling sites between REGICOR LUR and ESCAPE LUR was attributable to the conceptual differences in the modeling designs. Although REGICOR was aimed at capturing the small-scale variation between residential addresses of cohort members in a Mediterranean city with narrow street canyons, ESCAPE was aimed at capturing exposure to main emission sources in a standardized manner all across regions in Europe. Comparison of performance between the REGICOR and the ESCAPE LUR models has been evaluated elsewhere (de Nazelle et al. 2013). This study showed that models performed relatively similarly well at predicting their own measured concentrations, but the ESCAPE model increasingly overpredicted the measurements of independent data sets at higher NO_2_ levels. We found that for the same contrast of 10 μg/m^3^ in exposure to NO_2_, Rivera et al. (2013) reported a 0.22% (95% CI: –2.24%, 2.74%) coefficient for CIMT compared with –0.18% (95% CI: –0.89%, 0.53%) in our study. It has also been shown that the number of predictors tested to develop the LUR and the number of measurements influence the model performance (Basagaña et al. 2012, 2013; Wang et al. 2012, 2013). This may have also contributed to some nondifferential biases in the ESCAPE model. In HNR, past individual exposure to PM_2.5_ was the average of daily concentrations of the 365 days before the examination day (Bauer et al. 2010). PM_2.5_ individual exposures were predicted by a chemistry transport model coupled with daily data from monitoring stations (European Air Pollution Dispersion Model; EURAD-CTM). Again, the concepts of the exposure models differed between ESCAPE and the original HNR study, for which the EURAD-CTM exposure modeling was aimed at capturing urban background particulate matter concentrations (1-km^2^ grid). Estimates reported by Bauer et al. (2010) correspond to a 4.9% (95% CI: 2.0%, 7.7%) difference in CIMT per 5 μg/m^3^ PM_2.5_, whereas our estimate was 0.57% (95% CI: –1.95%, 3.14%) for the same exposure contrast. There remains a need to better understand bias from the different exposure models and implications for interpreting and comparing findings from epidemiological studies.

In a meta-analysis of four new cross-sectional European studies developed under standardized exposure and analytical protocols, we found no significant associations between CIMT and long-term estimates of residential exposure to eight predefined markers of air pollution, namely PM_2.5_, PM_2.5abs_, traffic load within 100 m of home, and traffic intensity at the nearest road. This contrasts with the strong experimental evidence for an atherogenic role of ambient particulate matter (Araujo and Nel 2009; Sun et al. 2005; Suwa et al. 2002). Our meta-analytic estimate across all published studies for CIMT and PM_2.5_ was suggestive but not statistically significant. Given the public health relevance of atherosclerosis, further studies are needed to clarify the quantitative association between markers of atherogenesis and long-term exposure to air pollution and both the cross-sectional level and the longitudinal progression of atherosclerosis.

**Editor’s Note:** After Advance Publication of this article, Perez et al. added Maria Foraster as a co-author and corrected the misspelling of co-author Damiano Baldassare to Damiano Baldassarre.The authors regret the error.

## Supplemental Material

(1.7 MB) PDF
