# Supplementary material for: Air Pollution and Atherosclerosis: A Cross-Sectional Analysis of Four European Cohort Studies in the ESCAPE Study
Source: Environ Health Perspect. 2015 Jan 27;123(6):597–605. doi: 10.1289/ehp.1307711 (PMC4455580; doi:10.1289/ehp.1307711)
Supplement: (1.7 MB) PDF [file ehp.1307711.s001.508.pdf]

Supplemental Material

Air Pollution and Atherosclerosis: A Cross-Sectional Analysis of Four European Cohort Studies in the ESCAPE Study

Laura Perez, Kathrin Wolf, Frauke Hennig, Johanna Penell, Xavier Basagaña, Maria Foraster, Inmaculada Aguilera, David Agis, Rob Beelen, Bert Brunekreef, Josef Cyrys, Kateryna B. Fuks, Martin Adam, Damiano Baldassarre, Marta Cirach, Roberto Elosua, Julia Dratva, Regina Hampel, Wolfgang Koenig, Jaume Marrugat, Ulf de Faire, Göran Pershagen, Nicole M. Probst-Hensch, Audrey de Nazelle, Mark J. Nieuwenhuijsen, Wolfgang Rathmann, Marcela Rivera, Jochen Seissler, Christian Schindler, Joachim Thiery, Barbara Hoffmann, Annette Peters, and Nino Künzli

Description of cohorts and Carotid-Intima Media Thickness data collection

IMPROVE-Stockholm: This study is a subcohort of a 60 year olds cohort initiated in Sweden for identification of biological and socio-economic risk factors and predictors for cardiovascular diseases (CVD) through a cross-sectional health screening study. From August 1997 to March 1999, a random sample of every third man and woman living in Stockholm County, who was born between 1 July 1937 and 30 June 1938, was invited to the 60 year olds study. Having at least three risk factors for atherosclerosis was the inclusion criteria, having earlier life threatening or cardiovascular diseases were exclusion factors. Carotid ultrasound was performed using a Technos system (Esaote, Genoa, Italy), equipped with a 5-10 Mhz linear array probe. A total of 30 artery measurements for each participant were obtained. This consisted in obtaining scans of 3 segments by 3 projections for left and right common carotid artery (in its entire length), in addition to 1 segment by 3 projections for left and right bifurcation and internal carotid artery. Carotid- Intima Media Thickness (CIMT) was measured at each scan by semi-automatic edge detection system. A total of 529 participants from IMPROVE-Stockholm cohort with valid CIMT and exposure levels were available for the ESCAPE study.

Heinz Nixdorf Recall Study (HNR): The Heinz Nixdorf Recall Study is an ongoing population-based, cardiovascular cohort study that started in 2000 and included 4814 randomly selected participants aged 45 to 75 years from three large adjacent cities (Essen, Mülheim, Bochum) of the densely populated and highly industrialized Ruhr Area, Germany. A separate random sample from every city's mandatory population registry was drawn. After the baseline examination, participants were followed with a yearly questionnaire mailed to them. The first follow-up examination took place after 5 years. CIMT measurements were obtained by B-mode with VividFiVe; GE Ultrasound Europe via manual tracing. Measurements of the artery were

obtained from scans conducted at the left and right common carotid artery at the far artery wall in plaque-free area, approximately 10 mm proximal to the bulb. Per participant there were at least one and maximum 3 images retained at each side and less than 10 manual IMT measurements per subject and side conducted (0.1 cm intervals). Plaque formation defined as CIMT>50% of the adjacent CIMT was excluded from the measurements. A total of 4809 participants with valid Carotid- Intima Media Thickness and exposure levels were included in the ESCAPE study.

KORA: In the framework of the Cooperative Health Research in the Region of Augsburg (KORA), four surveys of inhabitants of the Augsburg region have been conducted since 1984.

The main objective of the baseline investigations was to assess health indicators (morbidity, mortality) and health care (utilization, costs), quantify prevalence of risk factors for cardiovascular and other chronic diseases, and study the impact of lifestyle, metabolic and genetic factors on cardiovascular and other chronic diseases. The two cross-sectional baseline population-representative surveys were conducted in 1994-1995 (survey S3) and 1999-2001 (survey S4) in the city of Augsburg and two adjacent rural counties. Age at baseline was between 45 and 74. Follow-up examinations of survey S3 and S4 participants were carried in 2004-2005 (F3) and 2006-2008 (F4). This study used CIMT data collected from the F4 survey. CIMT measurements were obtained via B-mode ultrasound (Sonoline G, Siemens Medicyal Solutions, Münich, Germany) with automatic tracing. Measurements of the artery included obtaining scans from the far wall of the left and right common carotid artery measured 10-15 mm from bulb at five different angles. The mean CIMT of the 3 best projections per left and right common carotid

artery was calculated. A total of 2738 participants with valid CIMT and exposure levels were available for the ESCAPE study.

REGICOR: Registre Gironí del Cor (REGICOR) is a population-based cohort within 10 individual communities of the Girona area in North-east of Spain. The cohort was enrolled in 1995, and there has been a follow-up in 2000, 2005, and 2007. Age at baseline was between 25 and 74. The last follow-up study which took place between 2007-2009, was specifically designed to collect CIMT measurements and other health and residential information. CIMT measurements were obtained by B-mode with the machine Acuson Aspen ultrasound via manual tracing. Scan protocols included collecting a total of 3 segments measurement of the artery per side. One image was taken on the common carotid artery, measured 10 mm proximal to the bulb, one image was taken on the carotid bulb (at the arterial far wall between the carotid dilatation and the carotid flow divider), and one image was taken from the interior carotid at 1 cm distal of the carotid flow divider. One IMT measurement per scan was conducted. A total of 2713 participants with valid CIMT were available for the ESCAPE study. For this cohort, several participants were assigned an exposure value for NO_2 of zero because of negative LUR predictions. We retained for our analysis only participants with individual NO_2 measurements above 10 $\mu g/m^3$ (N=2291).

Exposure assessment methods

The traffic indicators used in ESCAPE are traffic intensity on the nearest road (vehicles*day^{-1}) and traffic load on major roads in a 100-meter buffer, defined as the sum of traffic intensity multiplied by the length of all major road segments (vehicles*meters^{-1}*day^{-1}). These traffic indicators were developed with GIS linking traffic counts to street maps, although the approach

differed depending on the local data available. In general, for motorways actual traffic counts were used as those are available across most areas in Europe. For other roads, if counts were not available, traffic forecast models were used or flow was estimated from counts. This has been previously described in (Eeftens et al. 2012). In brief, the source of traffic data for our 4 cohorts were as follow:

IMPROVE in the Stockholm region, Sweden: Traffic data is from a local road network with linked traffic intensities (mvh/24h) with 90% of roads covered. Traffic intensities were attached to all included roads with intensities higher than 500 vehicles/ 24hrs within Stockholm County. Counts for year 1993 to 2008 were available.

KORA in the Münich/Ausburg area, Germany: Local road network based on Basic DLM in 2009 (Digital Landscape Model) for road traffic with linked road types and traffic counts. DLM covers the whole study area. Traffic counts of the Street Directorate are limited to regional streets, traffic counts of Münich and Augsburg Municipal works service and Environmental Agency are limited to urban streets. Missing traffic counts were estimated based on the 50th percentile of the observed values for the corresponding street type.

NHR in the Ruhr area, Germany: Local road network with road classes and daily traffic volume of vehicle classes only on 50,000 road segments in North Rhine Westphalia for year 2007.

REGICOR in Catalunya, Spain: Local road network with linked traffic intensities (mvh/24h) in 2007. Map was only for roads with counts >5,000. Since the map was not complete for small roads it was only used for calculating variables related to major roads (distinvmajor, trafmajor, trafmajorload_X).

Table S1. Distribution of exposure to selected categories of two traffic indicators over full cohort with valid exposure and CIMT measurements.

**Exposure category**	**IMPROVE–Stockholm (N=529)**	**HNR (N=4809)**	**KORA (N=2738)**	**REGICOR (N=2291)**
Traffic intensity at the nearest road ($veh*day^{-1}$)				
<1,000	81.9%	NA	84.3%	46%
1,000-5,000	11.1%	NA	7.2%	33.3%
5,000-10,000	4.2%	NA	5.2%	11%
>10,000	2.8%	NA	3.3%	9.6%
Traffic load within 100m on major roads ($veh*day^{-1}*m^{-1}$)				
<500,000	81.9%	65.0%	78.7%	56.6%
500,000-1,500,000	0%	0%	17.1%	0%
1,500,000-3,000,000	15.3%	24.0%	0%	25.8%
>300,000,000	2.8%	11.0%	4.1%	16.3%

NA: not available.

Table S2. Spearman correlation coefficients (r) across individually assigned pollutant levels and traffic indicators[a] and previously published coefficient of variance (R^2) for ESCAPE land use regression model validation (Beelen et al. 2013; Eeftens et al. 2012).

Cohort/exposure	$PM_{2.5}$	PM_{25abs}	PM_{coarse}	PM_{10}	NO_2	NO_x	Traffic intensity	Validation model: No. sites	R^2	LOOCV[b] R^2	Difference
**IMPROVE-Stockholm (n=487)**											
$PM_{2.5}$	--	--	--	--	--	--	--	19	85%	78%	7%
$PM_{2.5abs}$	0.85	--	--	--	--	--	--	19	89%	85%	4%
PM_{coarse}	0.62	0.63	--	--	--	--	--	19	72%	65%	7%
PM_{10}	0.62	0.63	1.00	--	--	--	--	19	84%	77%	7%
NO_2	0.63	0.89	0.60	0.60	--	--	--	39	88%	83%	5%
NO_x	0.57	0.79	0.55	0.55	0.94	--	--	39	90%	87%	3%
Traffic intensity	0.47	0.48	0.48	0.53	0.58	0.47	--	--	--	--	--
Traffic load	0.48	0.64	0.46	0.46	0.62	0.52	0.39	--	--	--	--
**HNR (n=3759)**											
$PM_{2.5}$	--	--	--	--	--	--	--	20	88%	79%	9%
$PM_{2.5abs}$	0.88	--	--	--	--	--	--	20	97%	95%	2%
PM_{coarse}	0.68	0.72	--	--	--	--	--	20	66%	57%	9%
PM_{10}	0.88	0.89	0.69	--	--	--	--	20	69%	63%	6%
NO_2	0.65	0.62	0.46	0.54	--	--	--	40	89%	84%	5%
NO_x	0.62	0.52	0.42	0.51	0.93	--	--	40	88%	76%	12%
Traffic intensity	--	--	--	--	--	--	--	--	--	--	--
Traffic load	0.21	0.39	0.2	0.21	0.56	0.39	--	--	--	--	--
**KORA (n=2646)**											
$PM_{2.5}$	--	--	--	--	--	--	--	20	78%	62%	16%
$PM_{2.5abs}$	0.44	--	--	--	--	--	--	20	91%	82%	9%
PM_{coarse}	0.28	0.83	--	--	--	--	--	20	81%	69%	12%
PM_{10}	0.39	0.67	0.77	--	--	--	--	20	83%	75%	8%
NO_2	0.38	0.66	0.79	0.67	--	--	--	40	86%	67%	19%
NO_x	0.41	0.75	0.85	0.69	0.92	--	--	40	88%	81%	7%
Traffic intensity	0.20	0.20	0.20	0.14	0.27	0.26	--		--	--	
Traffic load	0.28	0.42	0.33	0.27	0.37	0.39	0.34		--	--	
**REGICOR (n=2291)**											
$PM_{2.5}$	--	--	--	--	--	--	--	40	62%	51%	11%
$PM_{2.5abs}$	0.84	--	--	--	--	--	--	40	75%	69%	6%
PM_{coarse}	0.12	0.11	--	--	--	--	--	40	76%	71%	5%
PM_{10}	0.39	0.45	0.46	--	--	--	--	40	76%	71%	5%
NO_2	0.67	0.85	0.09	0.5	--	--	--	80	71%	69%	2%
NO_x	0.7	0.88	0.15	0.56	0.99	--	--	80	69%	66%	3%
Traffic intensity	0.55	0.6	0.08	0.37	0.44	0.50	--	--	--	--	
Traffic load	0.35	0.38	0.45	0.38	0.33	0.36	0.29	--	--	--	

[a]Traffic intensity: traffic intensity at the nearest road; traffic load: traffic load within 100m on major roads. [b]LOOCV: leave one out cross validation.

Table S3. Meta-analysis of the association (with 95% Confidence Intervals) between carotid intima-media thickness (CIMT, geometric mean) and categories of traffic indicators for Model M3 additionally adjusted with background levels of NO_2. Effect estimates represent percent difference in CIMT between the relevant traffic category and the reference group. N is number of cohorts included in the meta-analysis. Heterogeneity across studies is represented by I^2 and p value for heterogeneity.

**Indicator**	**N**	**Percent difference[a] (95%CI)**	**p value**	**I^2**	**p heterogeneity**
Traffic intensity at the nearest road ($veh*day^{-1}$)[b]					
<1,000	Ref	Ref	NA	NA	NA
1,000-5,000	3	0.63% (–0.94, 2.23%)	0.432	37%	0.207
5,000-10,000	3	0.04% (–1.39, 1.49%)	0.956	0%	0.803
>10,000	3	0.86% (–0.94, 2.69%)	0.349	6%	0.347
Traffic load within 100m on major roads ($veh*day*m^{-1}$)					
<500,000	Ref	Ref	NA	NA	NA
500,000-1,500,000[c]	NA	NA	NA	NA	NA
1,500,000-3,000,000[d]	3	0.7% (–1.11, 2.54%)	0.453	50%	0.133
>3,000,000	4	0.13% (–1.17, 1.44%)	0.851	20%	0.291

NA: not available or not applicable. Ref: reference level. Model M3 adjusted for: sex, age (centered), age^2, smoking status (3 categories), smoking pack years (centered), smoking $pack\text{-}years^2$, education level (3 categories), occupation status (4 categories), BMI (centered), BMI^2, indicator of city residence when applying.

[a]Percent difference derived over CIMT geometric mean difference. [b]Analysis over three cohorts only, traffic intensity data is not available for HNR. [c]No meta-analysis conducted for this category as there was a zero count for three out of the four cohorts (IMPROVE-Stockholm, HNR, and REGICOR). [d]KORA not included in the meta-analysis for this category due to zero count.

Table S4. Meta-analysis of the association (with 95% Confidence Intervals) between carotid intima-media thickness (CIMT, geometric mean) and air pollution for Model M3 under different model parameters modification. Effect estimates represent percent difference in CIMT per standard contrast of exposure. Random-effects only reported. N is number of cohorts included in the meta-analysis and n the sample size. Heterogeneity across studies is represented by I^2 and p value for heterogeneity.

Model exposure	Contrast exposure	N	n	Percent difference[a] (95%CI)	p value	I^2(%)	p heterogeneity
**Random effects, neighborhood[b]**							
$PM_{2.5}$ (µg/m^3)	5	4	9178	0.69% (-0.89, 2.29%)	0.395	14	0.320
$PM_{2.5abs}$ (10^{-5}xm^{-1})	1	4	9178	0.49% (-1.44, 2.45%)	0.623	50	0.112
PM_{coarse} (µg/m^3)	10	4	9178	-0.67% (-4.8, 3.63%)	0.756	73	0.011
PM_{10} (µg/m^3)	10	4	9178	-0.78% (-3.52, 2.05%)	0.586	70	0.017
NO_2 (µg/m^3)	10	4	9178	-0.49% (-1.98, 1.03%)	0.526	63	0.044
NO_x (µg/m^3)	20	4	9178	-0.3% (-1.63, 1.06%)	0.668	62	0.048
Traffic intensity at the nearest road (veh/dayx10^{-5})	5,000	3[c]	5419	0.04% (-0.79, 0.88%)	0.922	54	0.110
Traffic load within 100m on major roads (veh/dayxmx10^{-5})	4,000,000	4	9178	0.87% (-0.72, 2.47%)	0.286	55	0.082
**Additional adjustment by noise (Lden dB)**							
$PM_{2.5}$ (µg/m^3)	5	4	7438	1.0% (-0.68, 2.7%)	0.244	0	0.461
$PM_{2.5abs}$ (10^{-5}xm^{-1})	1	4	7438	0.22% (-0.97, 1.43%)	0.713	0	0.441
PM_{coarse} (µg/m^3)	10	4	7438	-0.05% (-2.36, 2.3%)	0.964	0	0.861
PM_{10} (µg/m^3)	10	4	7438	-0.24% (-1.65, 1.18%)	0.736	0	0.826
NO_2 (µg/m^3)	10	4	7438	-0.97% (-2.04, 0.12%)	0.080	50	0.113
NO_x (µg/m^3)	20	4	7438	-0.56% (-1.29, 0.19%)	0.143	17	0.305
Traffic intensity at the nearest road (veh/dayx10^{-5})	5,000	3[c]	3738	-0.13% (-0.71, 0.46%)	0.674	0	0.408
Traffic load within 100m on major roads (veh/dayxmx10^{-5})	4,000,000	4	7438	1.79% (-2.79, 16.58%)	0.450	83	0.001
**Exposure back extrapolated to year of CIMT measurements[d]**							
PM_{10} (µg/m^3)	10	4	9183	-0.37% (-3.19, 2.52%)	0.797	79	0.003
NO_2 (µg/m^3)	10	4	9183	-0.68% (-1.87, 0.53%)	0.269	62	0.046
NO_x (µg/m^3)	10	4	9183	-0.19% (-0.66, 0.28%)	0.420	52	0.101

Model M3: sex, age (centered), age^2, smoking status (3 categories), smoking pack years (centered), smoking pack-years^2, education level (3 categories), occupation status (4 categories), BMI (centered), BMI^2, and indicator of city residence when applies.

[a]Percent difference over CIMT geometric mean difference. [b]The scale of the "neighbourhood" indicator considered for each cohort differed. IMPROVE-Stockholm and HNR: neighbourhood level; REGICOR: municipal level; KORA: indicator based on percentage of low income in 5x5 km grid. [c]Analysis over three cohort only, traffic intensity data was not available for HNR. [d]Individual exposure estimates replaced with estimates back-extrapolated to the year of the CIMT measurement. Back-extrapolated levels calculated as follows: in each study region, available historic annual means from fixed site monitoring stations were used to calculate the ratio between the average annual concentrations for the period of interest in the past and the period of the ESCAPE measurement. Individual ESCAPE exposure for each study participant was then multiplied by this ratio.

Figure S1. Percent difference in CIMT (geometric mean with 95% Confidence Intervals represented by bars) per standard contrast of exposure for (A) IMPROVE-Stockholm (B) HNR, (C) KORA, and (D) REGICOR, (E) Traffic intensity and (F) Traffic load. Percent change is calculated for the following contrast of exposure: 5 $\mu g/m^3$ for $PM_{2.5}$, 10 $\mu g/m^3$ for PM_{coarse}, PM_{10}, and NO_2, 20 $\mu g/m^3$ for NO_x and 1 x ($10^{-5}xm^{-1}$) for $PM_{2.5abs}$. Total population size represented by n. Adjustment sets are as follows: M1: crude; M2: sex, age; M3: sex, age (centered), age^2, smoking status (3 categories), smoking pack years (centered), smoking pack-$years^2$, education level (3 categories), occupation status (4 categories), BMI (centered), BMI^2, indicator of city residence; M4a: M3 + metabolic calories, alcohol per week (categorical), wine per week (centered), wine per $week^2$; M4b: M4a + systolic blood pressure, LDL, HDL; M5: M4b+ statin medication, and hypertension medication.

Figure 1.

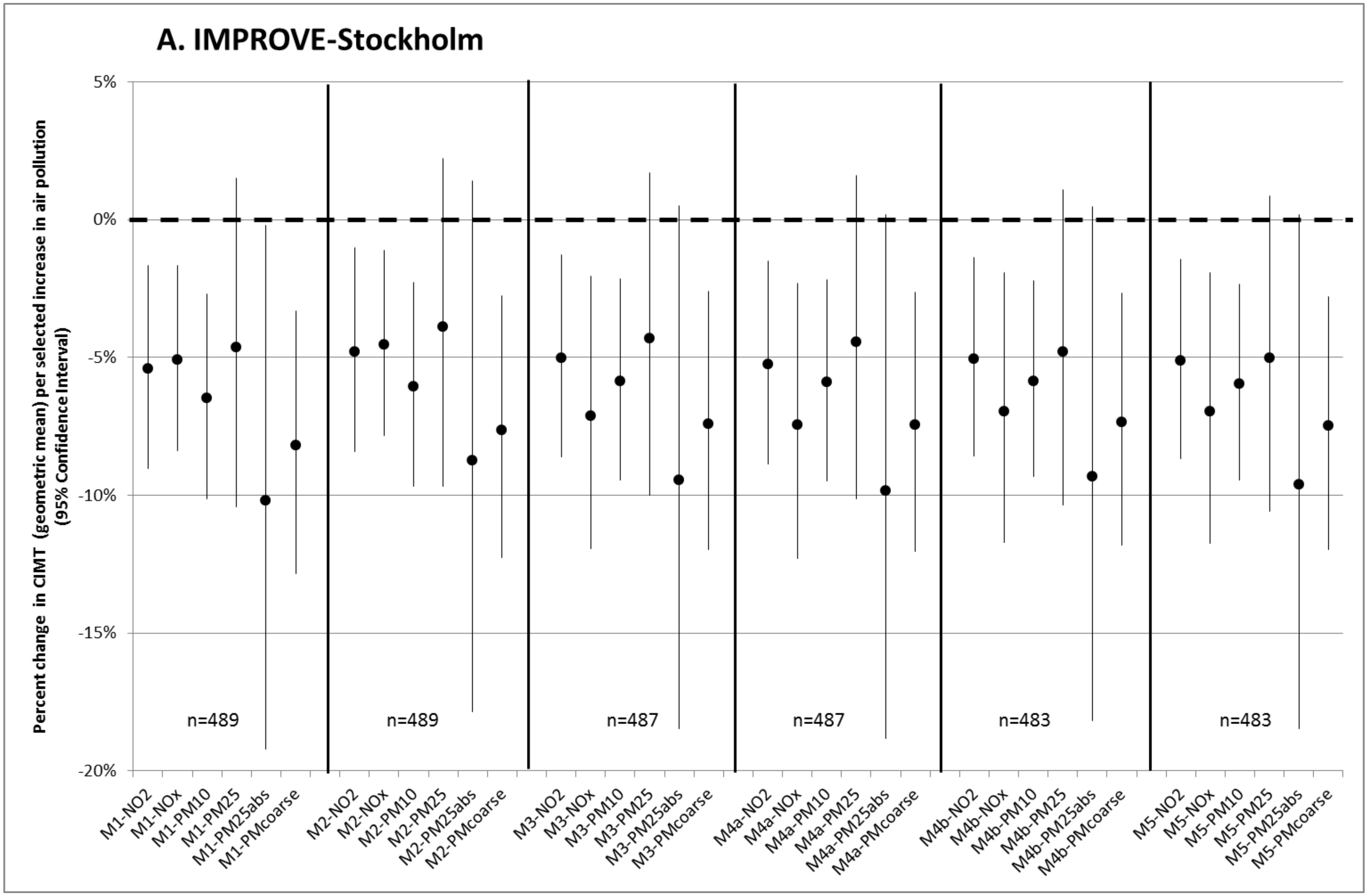
A. IMPROVE-Stockholm
Percent change in CIMT (geometric mean) per selected increase in air pollution (95% Confidence Interval)
5%
0%
-5%
-10%
-15%
-20%
n=489
n=489
n=487
n=487
n=483
n=483
M1-NO2
M1-NOx
M1-PM10
M1-PM25
M1-PM25abs
M1-PMcoarse
M2-NO2
M2-NOx
M2-PM10
M2-PM25
M2-PM25abs
M2-PMcoarse
M3-NO2
M3-NOx
M3-PM10
M3-PM25
M3-PM25abs
M3-PMcoarse
M4a-NO2
M4a-NOx
M4a-PM10
M4a-PM25
M4a-PM25abs
M4a-PMcoarse
M4b-NO2
M4b-NOx
M4b-PM10
M4b-PM25
M4b-PM25abs
M4b-PMcoarse
M5-NO2
M5-NOx
M5-PM10
M5-PM25
M5-PM25abs
M5-PMcoarse

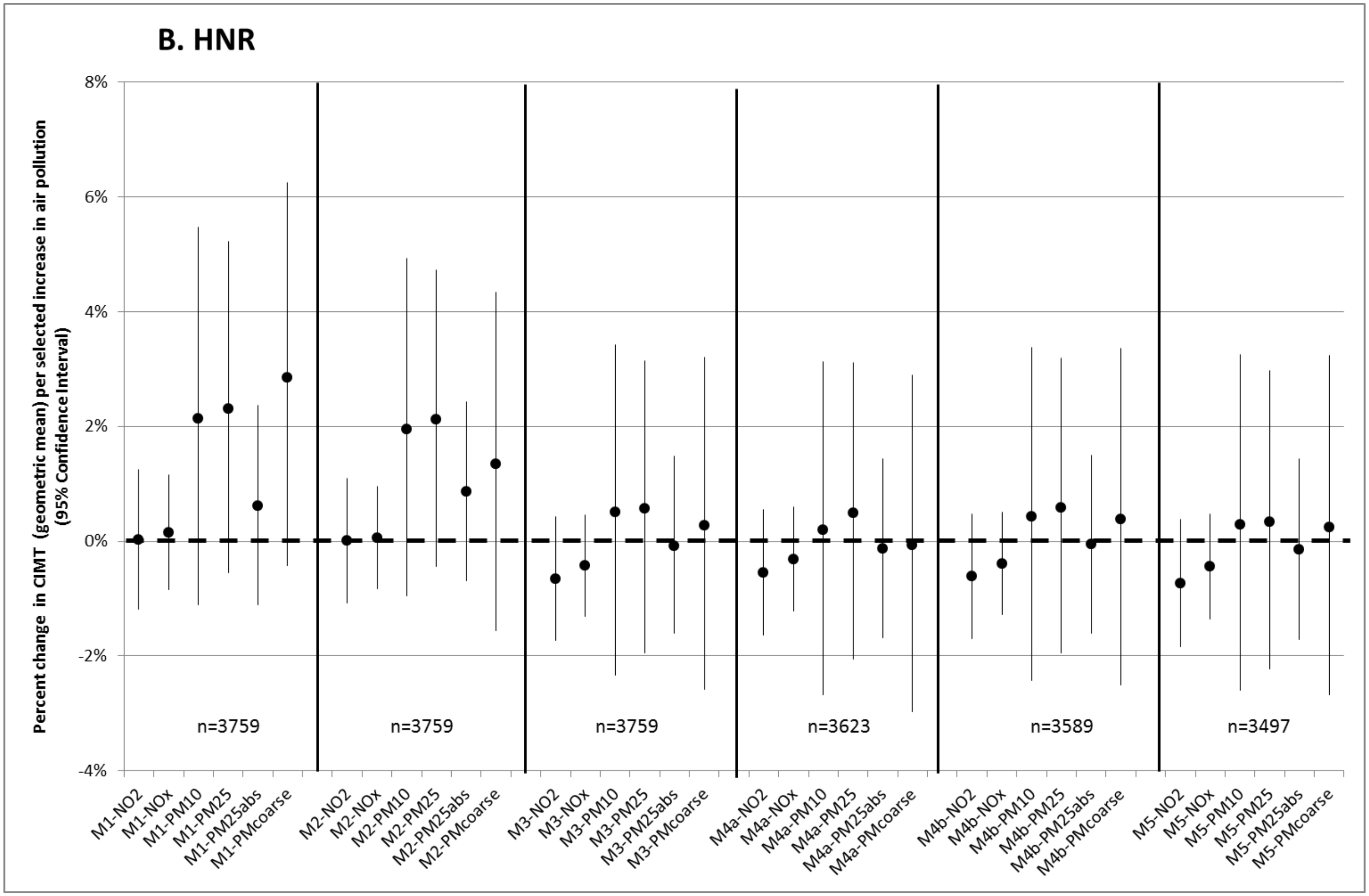
B. HNR
Percent change in CIMT (geometric mean) per selected increase in air pollution (95% Confidence Interval)
8%
6%
4%
2%
0%
-2%
-4%
n=3759
n=3759
n=3759
n=3623
n=3589
n=3497
M1-NO2
M1-NOx
M1-PM10
M1-PM25
M1-PM25abs
M1-PMcoarse
M2-NO2
M2-NOx
M2-PM10
M2-PM25
M2-PM25abs
M2-PMcoarse
M3-NO2
M3-NOx
M3-PM10
M3-PM25
M3-PM25abs
M3-PMcoarse
M4a-NO2
M4a-NOx
M4a-PM10
M4a-PM25
M4a-PM25abs
M4a-PMcoarse
M4b-NO2
M4b-NOx
M4b-PM10
M4b-PM25
M4b-PM25abs
M4b-PMcoarse
M5-NO2
M5-NOx
M5-PM10
M5-PM25
M5-PM25abs
M5-PMcoarse

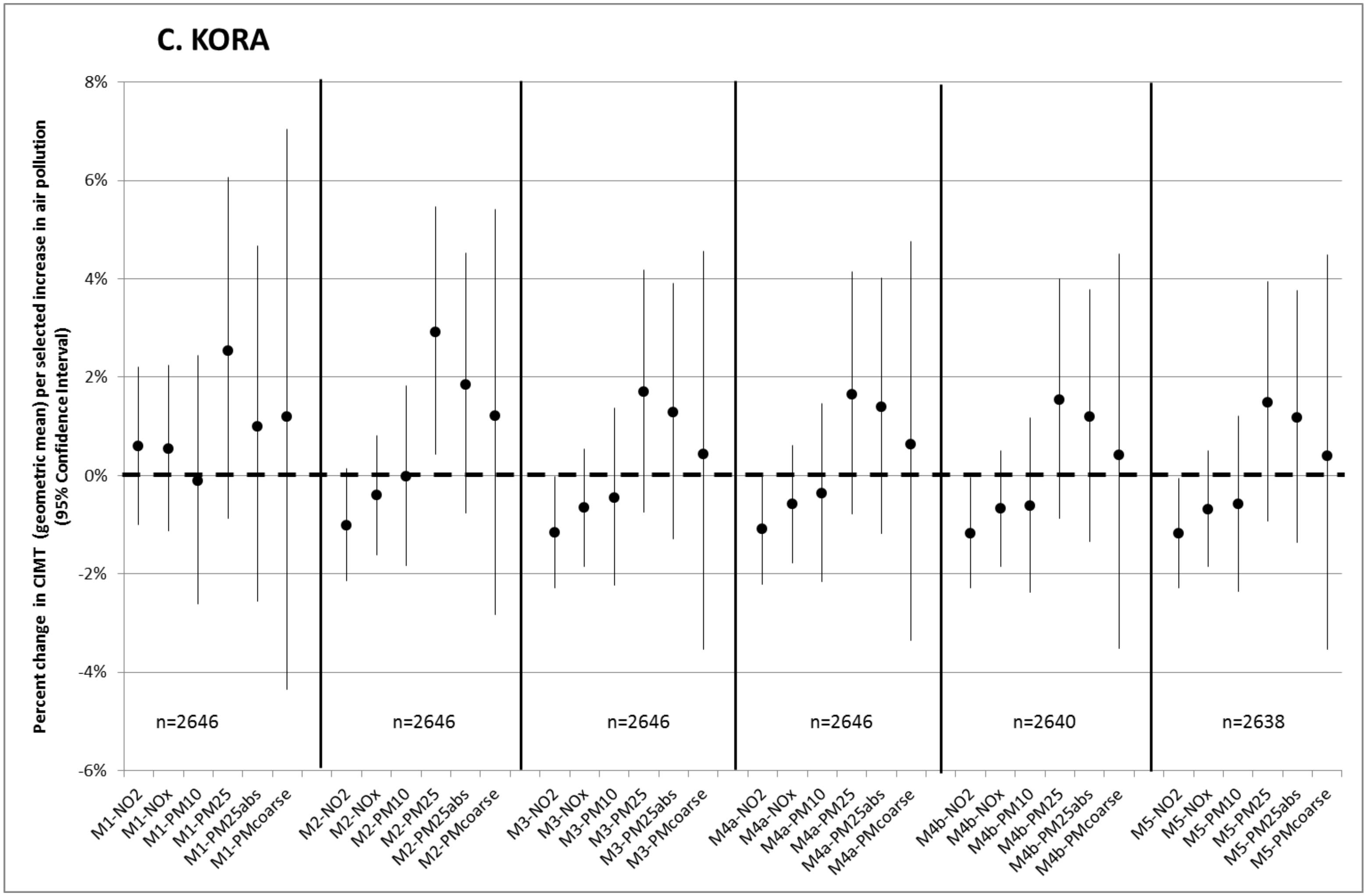
C. KORA
Percent change in CIMT (geometric mean) per selected increase in air pollution (95% Confidence Interval)
8%
6%
4%
2%
0%
-2%
-4%
-6%
n=2646
n=2646
n=2646
n=2646
n=2640
n=2638
M1-NO2
M1-NOx
M1-PM10
M1-PM25
M1-PM25abs
M1-PMcoarse
M2-NO2
M2-NOx
M2-PM10
M2-PM25
M2-PM25abs
M2-PMcoarse
M3-NO2
M3-NOx
M3-PM10
M3-PM25
M3-PM25abs
M3-PMcoarse
M4a-NO2
M4a-NOx
M4a-PM10
M4a-PM25
M4a-PM25abs
M4a-PMcoarse
M4b-NO2
M4b-NOx
M4b-PM10
M4b-PM25
M4b-PM25abs
M4b-PMcoarse
M5-NO2
M5-NOx
M5-PM10
M5-PM25
M5-PM25abs
M5-PMcoarse

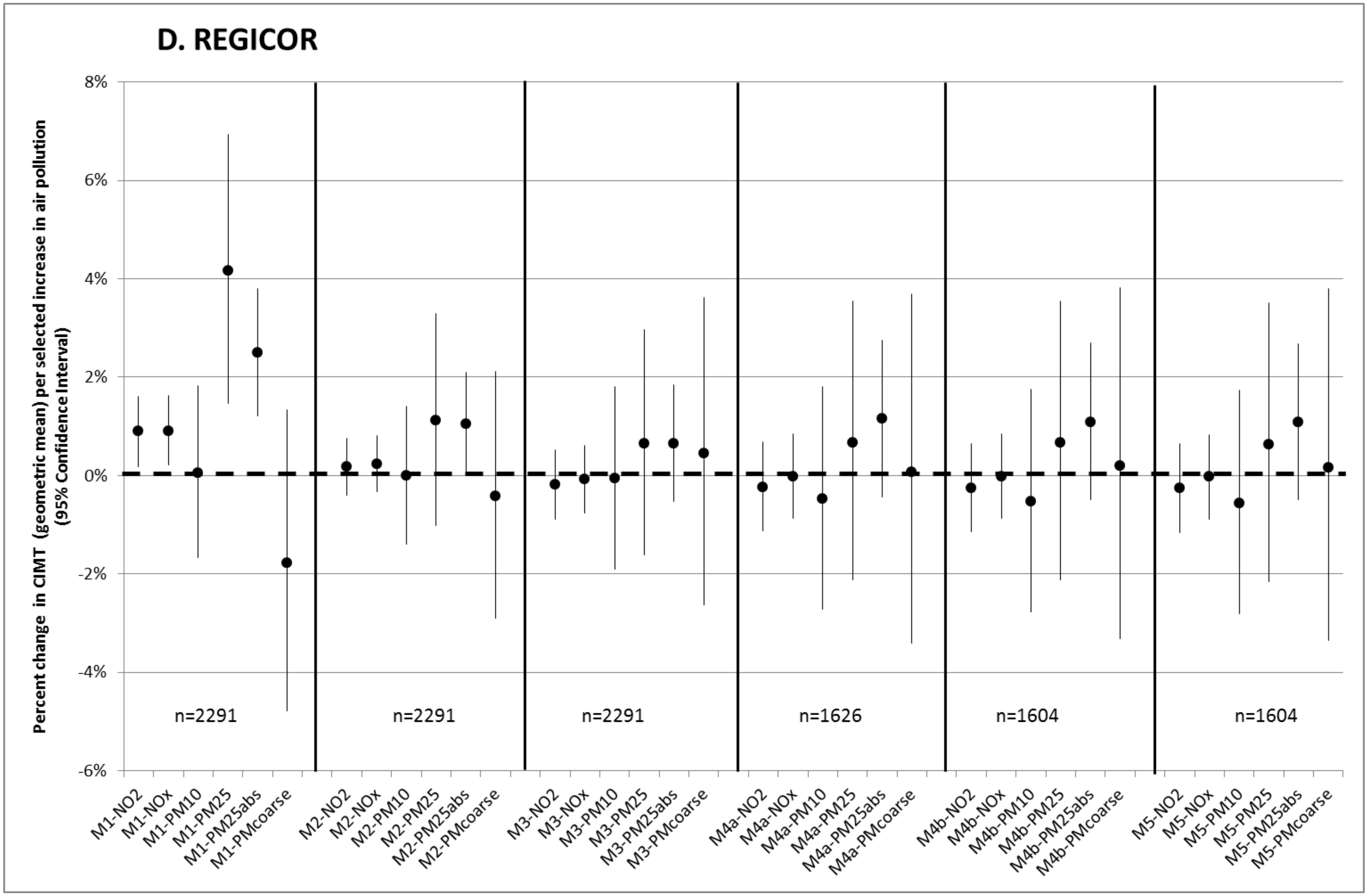
D. REGICOR
Percent change in CIMT (geometric mean) per selected increase in air pollution (95% Confidence Interval)
8%
6%
4%
2%
0%
-2%
-4%
-6%
n=2291
n=2291
n=2291
n=1626
n=1604
n=1604
M1-NO2
M1-NOx
M1-PM10
M1-PM25
M1-PM25abs
M1-PMcoarse
M2-NO2
M2-NOx
M2-PM10
M2-PM25
M2-PM25abs
M2-PMcoarse
M3-NO2
M3-NOx
M3-PM10
M3-PM25
M3-PM25abs
M3-PMcoarse
M4a-NO2
M4a-NOx
M4a-PM10
M4a-PM25
M4a-PM25abs
M4a-PMcoarse
M4b-NO2
M4b-NOx
M4b-PM10
M4b-PM25
M4b-PM25abs
M4b-PMcoarse
M5-NO2
M5-NOx
M5-PM10
M5-PM25
M5-PM25abs
M5-PMcoarse

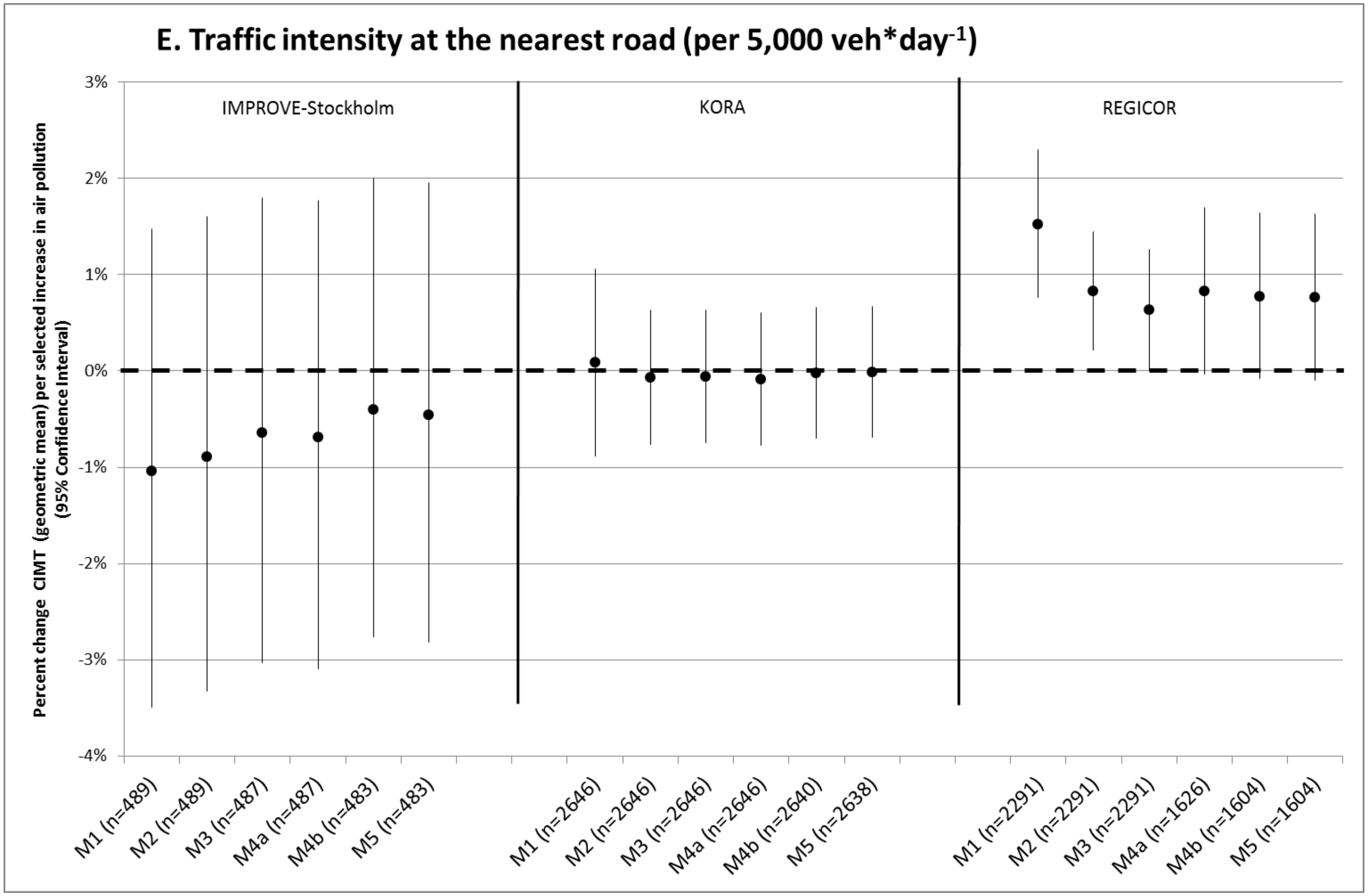
E. Traffic intensity at the nearest road (per 5,000 veh*day-1)
IMPROVE-Stockholm
KORA
REGICOR
Percent change CIMT (geometric mean) per selected increase in air pollution
(95% Confidence Interval)
3%
2%
1%
0%
-1%
-2%
-3%
-4%
M1 (n=489)
M2 (n=489)
M3 (n=487)
M4a (n=487)
M4b (n=483)
M5 (n=483)
M1 (n=2646)
M2 (n=2646)
M3 (n=2646)
M4a (n=2646)
M4b (n=2640)
M5 (n=2638)
M1 (n=2291)
M2 (n=2291)
M3 (n=2291)
M4a (n=1626)
M4b (n=1604)
M5 (n=1604)

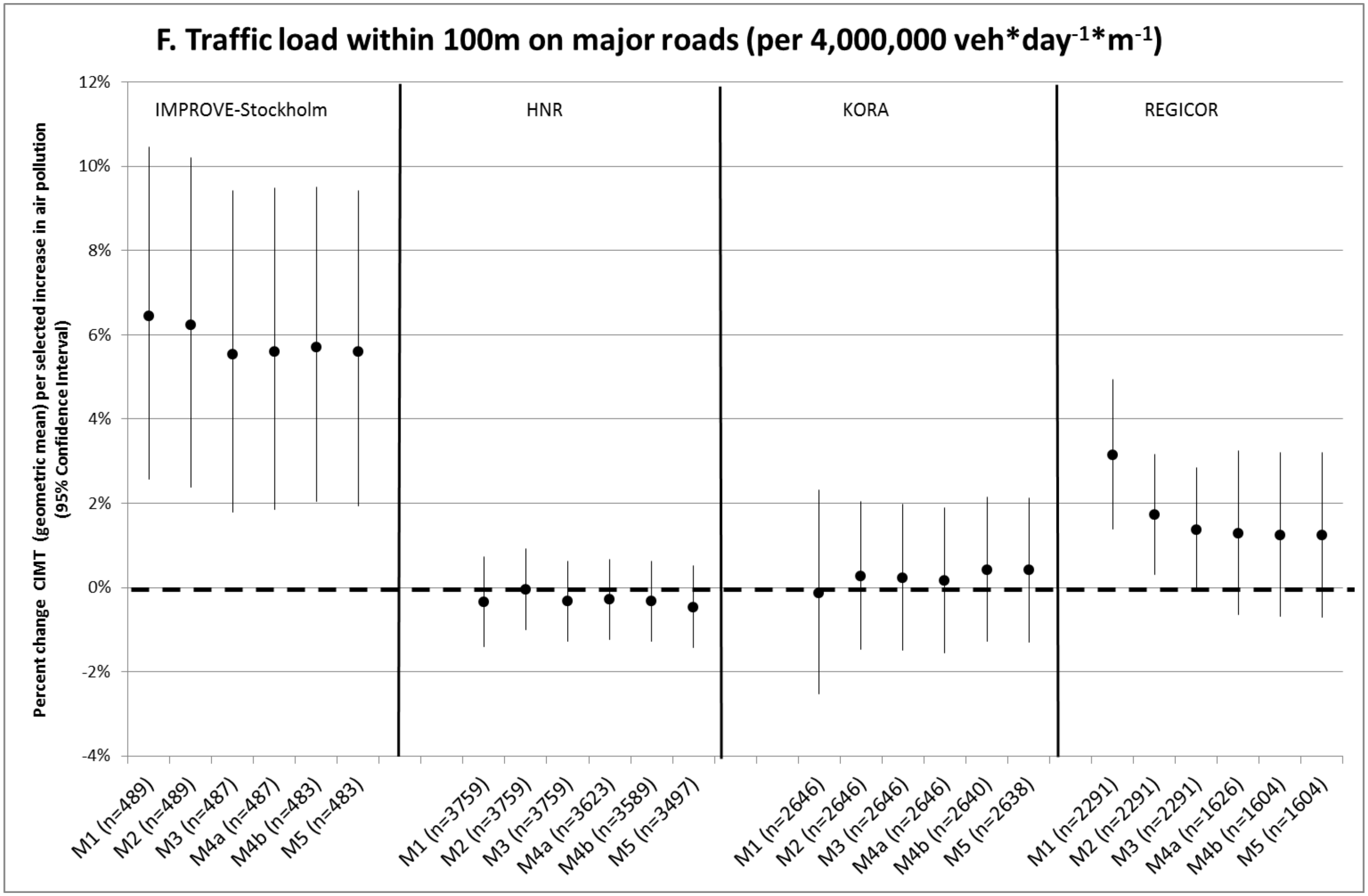
F. Traffic load within 100m on major roads (per 4,000,000 veh*day-1*m-1)
Percent change CIMT (geometric mean) per selected increase in air pollution (95% Confidence Interval)
12%
10%
8%
6%
4%
2%
0%
-2%
-4%
IMPROVE-Stockholm
HNR
KORA
REGICOR
M1 (n=489)
M2 (n=489)
M3 (n=487)
M4a (n=487)
M4b (n=483)
M5 (n=483)
M1 (n=3759)
M2 (n=3759)
M3 (n=3759)
M4a (n=3623)
M4b (n=3589)
M5 (n=3497)
M1 (n=2646)
M2 (n=2646)
M3 (n=2646)
M4a (n=2646)
M4b (n=2640)
M5 (n=2638)
M1 (n=2291)
M2 (n=2291)
M3 (n=2291)
M4a (n=1626)
M4b (n=1604)
M5 (n=1604)

Figure S2. Meta-analytic estimates of the association (with 95% Confidence Intervals) between CIMT (geometric mean) and (A) NO_x, (B) $PM_{2.5}$, and (C) $PM_{2.5abs}$ (Model M3). Percent difference in CIMT is calculated for the following contrast of exposure: 20 $\mu g/m^3$ for NO_x , 5 $\mu g/m^3$ for $PM_{2.5}$, and 1 x ($10^{-5}xm^{-1}$) for $PM_{2.5abs}$. N is number of cohorts included in the meta-analysis; n is the total population, based on individuals with no missings for any of these variables. Random-effects only reported, asterisks after label (*) represents a p value of heterogeneity below 0.05.

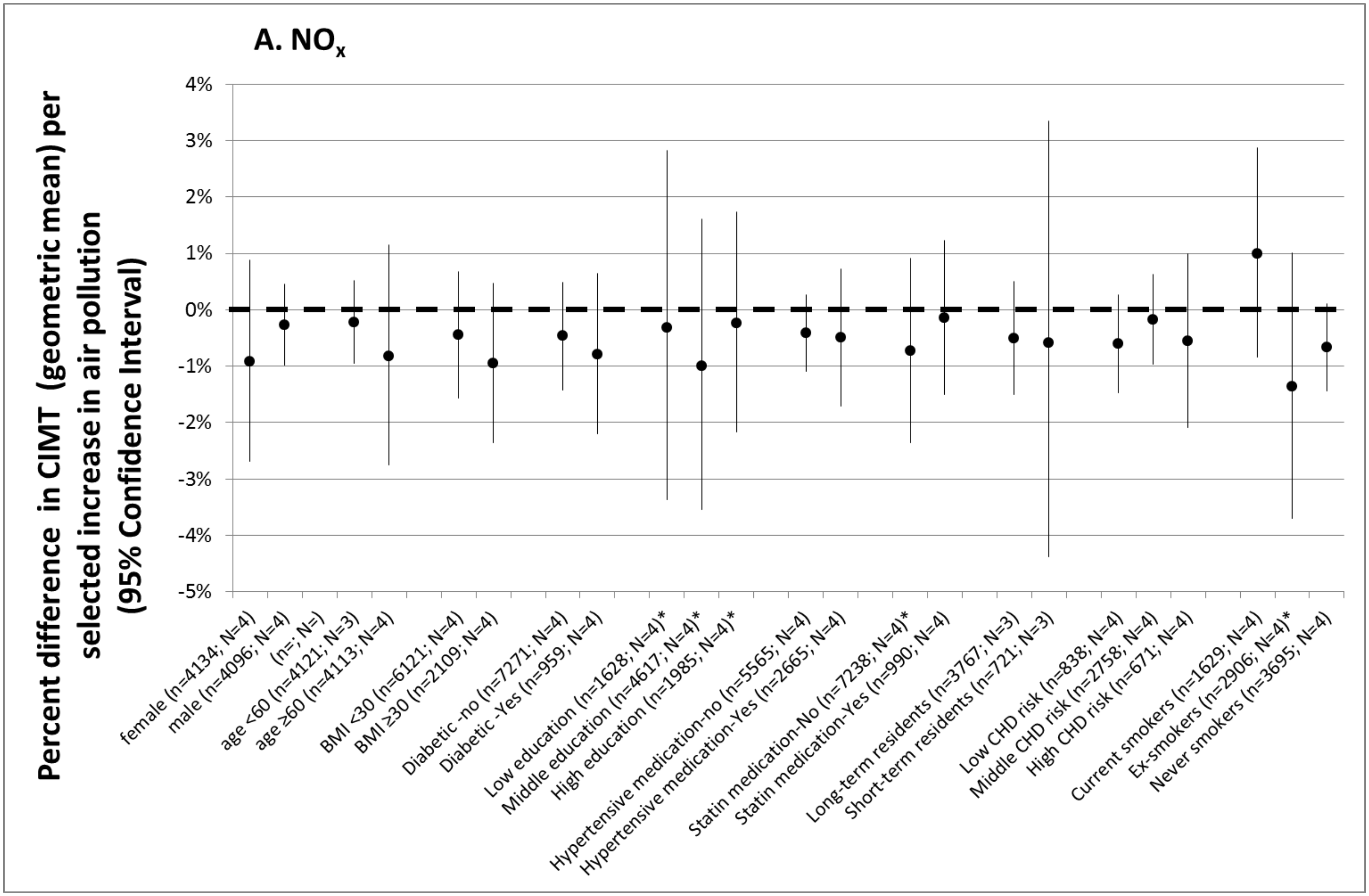
A. NO_x
Percent difference in CIMT (geometric mean) per selected increase in air pollution (95% Confidence Interval)
4%
3%
2%
1%
0%
-1%
-2%
-3%
-4%
-5%
female (n=4134; N=4)
male (n=4096; N=4)
(n=; N=)
age <60 (n=4121; N=3)
age ≥60 (n=4113; N=4)
BMI <30 (n=6121; N=4)
BMI ≥30 (n=2109; N=4)
Diabetic -no (n=7271; N=4)
Diabetic -Yes (n=959; N=4)
Low education (n=1628; N=4)*
Middle education (n=4617; N=4)*
High education (n=1985; N=4)*
Hypertensive medication-no (n=5565; N=4)
Hypertensive medication-Yes (n=2665; N=4)
Statin medication-No (n=7238; N=4)*
Statin medication-Yes (n=990; N=4)
Long-term residents (n=3767; N=3)
Short-term residents (n=721; N=3)
Low CHD risk (n=838; N=4)
Middle CHD risk (n=2758; N=4)
High CHD risk (n=671; N=4)
Current smokers (n=1629; N=4)
Ex-smokers (n=2906; N=4)*
Never smokers (n=3695; N=4)

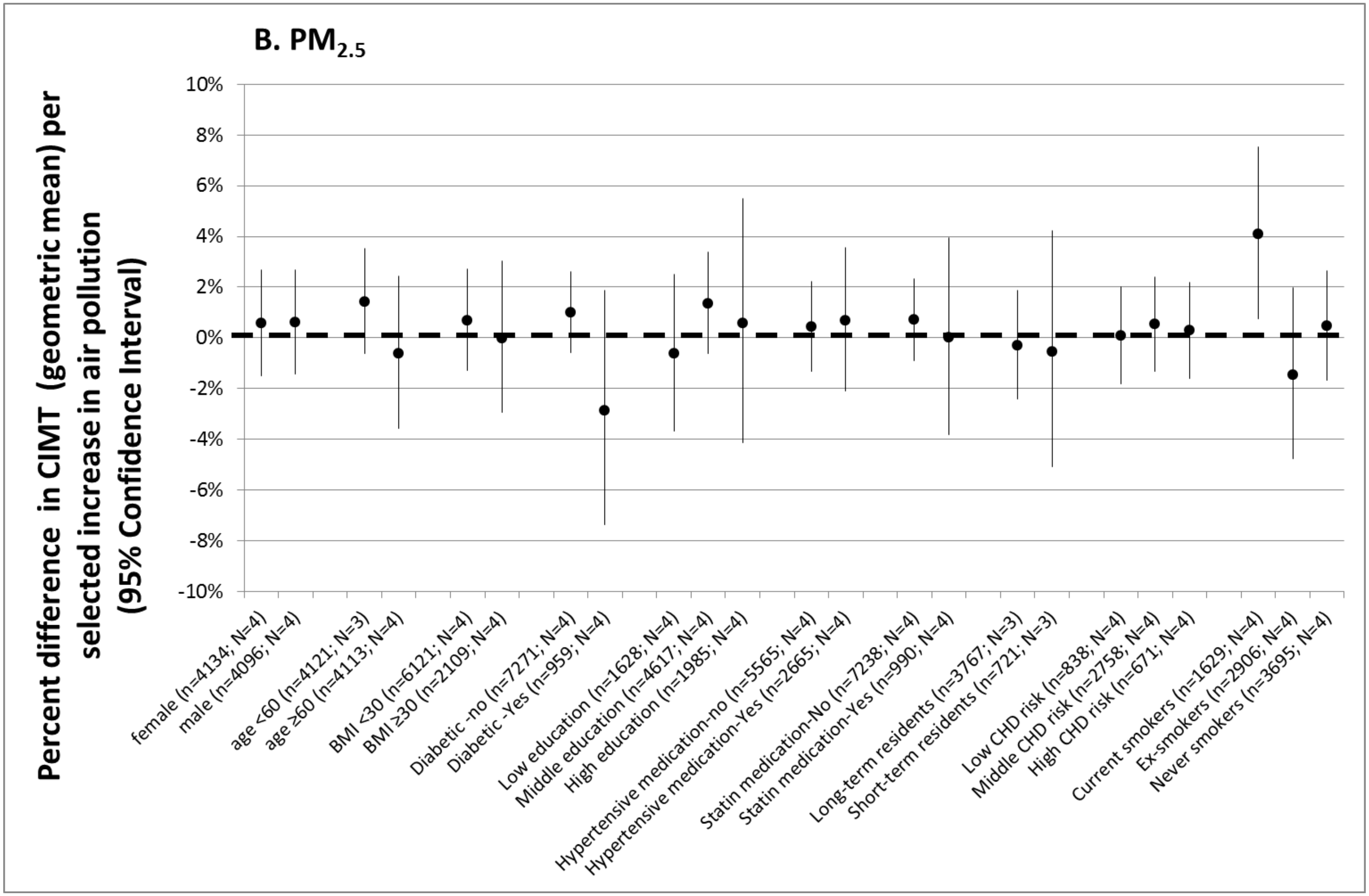
B. PM2.5
Percent difference in CIMT (geometric mean) per selected increase in air pollution (95% Confidence Interval)
10%
8%
6%
4%
2%
0%
-2%
-4%
-6%
-8%
-10%
female (n=4134; N=4)
male (n=4096; N=4)
age <60 (n=4121; N=3)
age ≥60 (n=4113; N=4)
BMI <30 (n=6121; N=4)
BMI ≥30 (n=2109; N=4)
Diabetic -no (n=7271; N=4)
Diabetic -Yes (n=959; N=4)
Low education (n=1628; N=4)
Middle education (n=4617; N=4)
High education (n=1985; N=4)
Hypertensive medication-no (n=5565; N=4)
Hypertensive medication-Yes (n=2665; N=4)
Statin medication-No (n=7238; N=4)
Statin medication-Yes (n=990; N=4)
Long-term residents (n=3767; N=3)
Short-term residents (n=721; N=3)
Low CHD risk (n=838; N=4)
Middle CHD risk (n=2758; N=4)
High CHD risk (n=671; N=4)
Current smokers (n=1629; N=4)
Ex-smokers (n=2906; N=4)
Never smokers (n=3695; N=4)

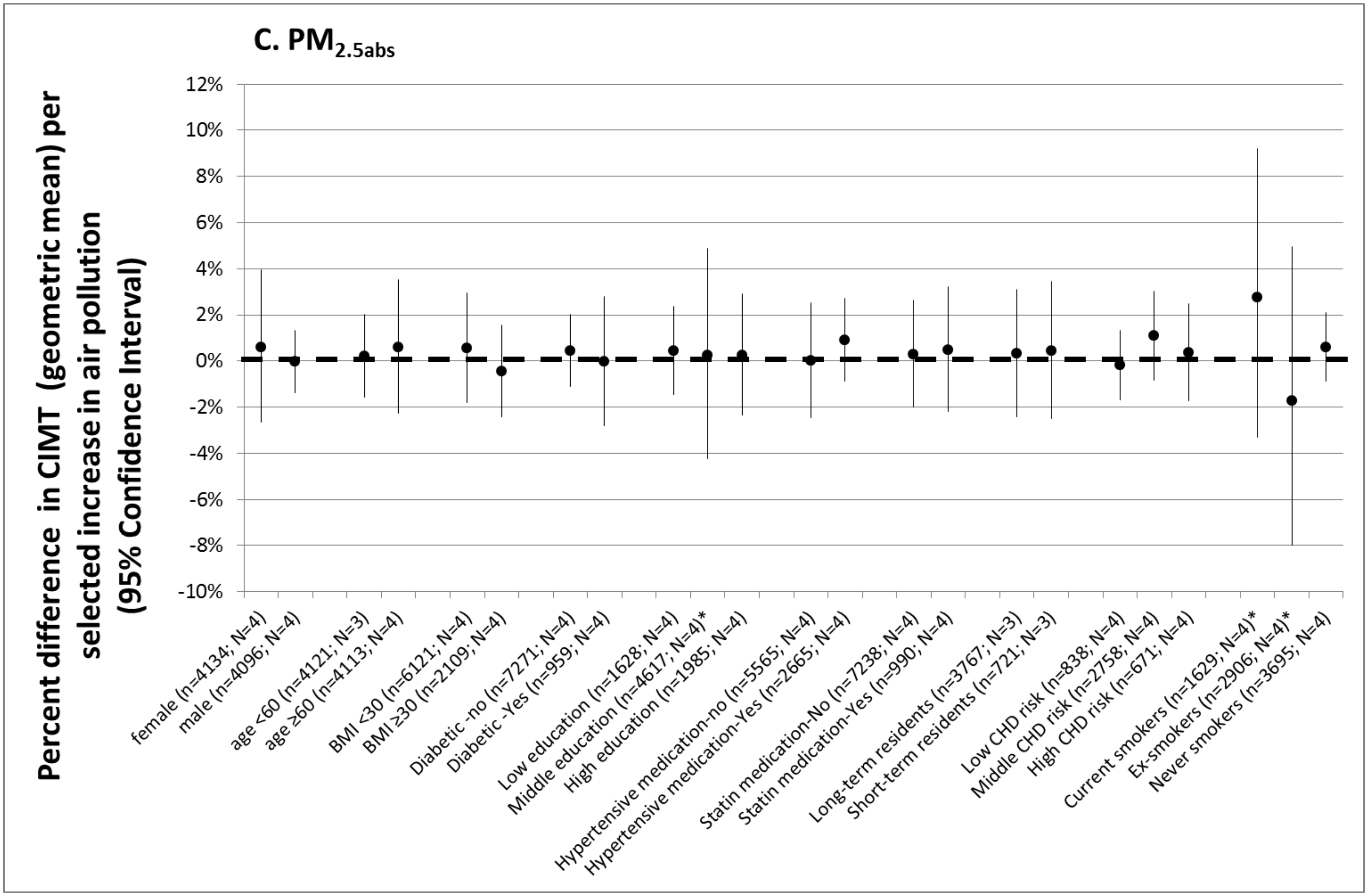
C. PM2.5abs
Percent difference in CIMT (geometric mean) per selected increase in air pollution (95% Confidence Interval)
12%
10%
8%
6%
4%
2%
0%
-2%
-4%
-6%
-8%
-10%
female (n=4134; N=4)
male (n=4096; N=4)
age <60 (n=4121; N=3)
age ≥60 (n=4113; N=4)
BMI <30 (n=6121; N=4)
BMI ≥30 (n=2109; N=4)
Diabetic -no (n=7271; N=4)
Diabetic -Yes (n=959; N=4)
Low education (n=1628; N=4)
Middle education (n=4617; N=4)*
High education (n=1985; N=4)
Hypertensive medication-no (n=5565; N=4)
Hypertensive medication-Yes (n=2665; N=4)
Statin medication-No (n=7238; N=4)
Statin medication-Yes (n=990; N=4)
Long-term residents (n=3767; N=3)
Short-term residents (n=721; N=3)
Low CHD risk (n=838; N=4)
Middle CHD risk (n=2758; N=4)
High CHD risk (n=671; N=4)
Current smokers (n=1629; N=4)*
Ex-smokers (n=2906; N=4)*
Never smokers (n=3695; N=4)

References

Beelen R, Hoek G, Vienneau D. 2013. Development of no2 and nox land use regression models for estimating air pollution exposure in 36 study areas in europe. The escape project. Atmospheric Environment 72 (2013) 10e23.

Eeftens M, Beelen R, de Hoogh K, Bellander T, Cesaroni G, Cirach M, et al. 2012. Development of land use regression models for pm(2.5), pm(2.5) absorbance, pm(10) and pm(coarse) in 20 european study areas; results of the escape project. Environmental science & technology 46:11195-11205.
